# Tomato locule number and fruit size controlled by natural alleles of *lc* and *fas*

**DOI:** 10.1002/pld3.142

**Published:** 2019-07-03

**Authors:** Yi‐Hsuan Chu, Jyan‐Chyun Jang, Zejun Huang, Esther van der Knaap

**Affiliations:** ^1^ Department of Horticulture and Crop Science The Ohio State University Wooster Ohio; ^2^ Department of Horticulture and Crop Science The Ohio State University Columbus Ohio; ^3^ Institute of Plant Breeding, Genetics and Genomics University of Georgia Athens Georgia; ^4^ Department of Horticulture University of Georgia Athens Georgia

**Keywords:** FASCIATED, fruit development, gene expression, LOCULE NUMBER, Tomato

## Abstract

Improving yield by increasing the size of produce is an important selection criterion during the domestication of fruit and vegetable crops. Genes controlling meristem organization and organ formation work in concert to regulate the size of reproductive organs. In tomato, *lc* and *fas* control locule number, which often leads to enlarged fruits compared to the wild progenitors. *LC* is encoded by the tomato ortholog of WUSCHEL (WUS), whereas *FAS* is encoded by the tomato ortholog of CLAVATA3 (CLV3). The critical role of the WUS‐CLV3 feedback loop in meristem organization has been demonstrated in several plant species. We show that mutant alleles for both loci in tomato led to an expansion of the *SlWUS* expression domain in young floral buds 2–3 days after initiation. Single and double mutant alleles of *lc* and *fas* maintain higher *SlWUS* expression during the development of the carpel primordia in the floral bud. This augmentation and altered spatial expression of *SlWUS* provided a mechanistic basis for the formation of multilocular and large fruits. Our results indicated that *lc* and *fas* are gain‐of‐function and partially loss‐of‐function alleles, respectively, while both mutations positively affect the size of tomato floral meristems. In addition, expression profiling showed that *lc* and *fas* affected the expression of several genes in biological processes including those involved in meristem/flower development, patterning, microtubule binding activity, and sterol biosynthesis. Several differentially expressed genes co‐expressed with *SlWUS* have been identified, and they are enriched for functions in meristem regulation. Our results provide new insights into the transcriptional regulation of genes that modulate meristem maintenance and floral organ determinacy in tomato.

## 1 INTRODUCTION

To develop varieties with improved characteristics, breeders exploit the genetic variation of important agronomic traits such as fruit weight, grain yield, and overall plant architecture. With the advent of Quantitative Trait Loci (QTL) mapping and cloning methods in past decades, many genes contributing to the increase in fruit weight and crop yield have been identified (Bommert, Nagasawa, & Jackson, 2013; Chakrabarti et al., 2013; Frary et al., 2000; Li et al., 2011; Song et al., 2007). The weight of fruits and vegetables is genetically controlled as early as inflorescence and floral meristem development (van der Knaap & Østergaard, 2017). Specifically, the organization of floral meristems is important in determining the final number of carpels, which collectively become the fruit after fertilization of the ovules (Fletcher, Brand, Running, Simon, & Meyerowitz, 1999; Mayer et al., 1998; Xu et al., 2015). Not only is fruit weight affected, the differential regulation of floral meristem development also accounts for variances in rice grain size and maize kernel number (Bommert et al., 2013; Suzaki et al., 2009). Together, these findings indicate that meristem‐regulated processes are essential and conserved features for reproductive organ development in higher plants.

Meristems maintain the balance between cell differentiation and self‐renewal in a coordinated manner through intercellular communication mediated by WUSCHEL (WUS) and CLAVATA3 (CLV3) proteins (Fletcher et al., 1999; Laux, Mayer, Berger, & Jurgens, 1996; Mayer et al., 1998). In Arabidopsis, CLV3 and WUS orchestrate meristem function via a negative feedback regulatory loop (Brand, Fletcher, Hobe, Meyerowitz, & Simon, 2000; Schoof et al., 2000). CLV3 signals are perceived directly or indirectly by different receptor complexes, principally CLV1, CLV2, CORYNE (CRN), BARELY ANY MERISTEM 1 (BAM1) and RECEPTOR‐LIKE PROTEIN KINASE 2 (RPK2) to restrict *WUS* expression (Shinohara & Matsubayashi, 2015; Somssich, Je, Simon, & Jackson, 2016). WUS activates *CLV3* expression at the meristem by binding to the *CLV3 cis*‐regulatory regions in its promoter (Perales et al., 2016; Yadav et al., 2011). CLV3 belongs to the CLE small peptide family and it acts in a non‐cell autonomous manner (Cock and McCormick 2001; Lenhard and Laux 2003). CLE family proteins typically share a putative N‐terminal signal peptide and the conserved CLE motif known to interact with CLV receptors (Ni & Clark, 2006; Rojo, Sharma, Kovaleva, Raikhel, & Fletcher, 2002; Shinohara & Matsubayashi, 2015). In addition, post‐translational modification of CLV3 and certain CLE peptides with a tri‐arabinoside chain is required to activate their functions through conformational changes (Shinohara & Matsubayashi, 2013; Xu et al., 2015). The null mutation in *clv3* leads to enlarged floral meristems due to the increase of the central zone, which contributes to the development of supernumerary floral organs (Brand et al., 2000; Fletcher et al., 1999). *WUS* encodes a homeodomain transcription factor required for specifying stem cell identity (Laux et al., 1996; Mayer et al., 1998). In Arabidopsis, the *wus* null mutant is characterized by aberrant meristem structure and premature termination of shoot apical meristems (SAMs) and floral meristems (FMs). Premature termination of FM leads to a restriction in stamen and carpel development (Laux et al., 1996). Principally, WUS positively regulates *CLV3* expression which in turn leads to downregulation of *WUS* expression through the interactions of CLV3 with membrane‐localized receptors and phosphorylation‐dependent downstream effectors (Betsuyaku, Takahashi et al., 2011; Brand et al., 2000; Schoof et al., 2000; Somssich et al., 2016; Song, Lee, & Clark, 2006). In addition to the CLV3‐WUS feedback loop, WUS also positively regulates the carpel identity gene, *AGAMOUS (AG),* during early floral development (Lenhard, Bohnert, Jürgens, & Laux, 2001; Lohmann et al., 2001). Subsequently, AG suppresses *WUS* expression by binding to the CArG box located downstream of the gene and the recruitment of polycomb group proteins, known to trigger transcriptional repression by enhancing histone methylation (Liu et al., 2011). An *ag* loss‐of‐function mutation completely abolishes carpel development in Arabidopsis (Yanofsky et al., 1990).

*LC* and *FAS* are two important genes contributing to enlarged fruits with many locules in tomato. Both mutants were selected among different genetic subgroups during tomato domestication because of their positive effects on fruit weight (Blanca et al., 2015; Rodríguez et al., 2011). However, a mutation in *FAS* often results in unacceptable fruits that are unevenly shaped and therefore, the allele is not commonly found in conventional and commercially grown tomatoes. *lc* is a mutation near *SlWUS*, and is expected to cause increased expression by abolishing the binding site of its suppressor AGAMOUS. The causative mutation is comprised of two SNPs that are downstream of the 3′ UTR of *SlWUS* (Muños et al., 2011). *fas* on the other hand is caused by a ~294 kb inversion with a breakpoint in the promoter region of *SlCLV3* (Huang & van der Knaap, 2011; Xu et al., 2015). Although the role of CLV3‐WUS in meristem maintenance has been investigated in Arabidopsis and other plant species, it remains unclear whether novel factors are involved in FM regulation in tomato. To understand the molecular mechanism underpinning LC‐FAS mediated developmental processes, we conducted a series of genetic and gene expression analyses using backcross populations (Figure S1). We show that *lc* is a gain‐of‐function mutation of *SlWUS*, whereas *fas* is a partial loss‐of‐function mutation of *SlCLV3*. Importantly, our RNA‐seq results led to the identification of a number of co‐expressed gene clusters associated with various developmental processes that might act downstream of *LC* and *FAS*.

## 2 RESULTS

### 2.1 The effects of *lc* and *fas* on fruit morphology and reproductive traits

**Figure 1:**
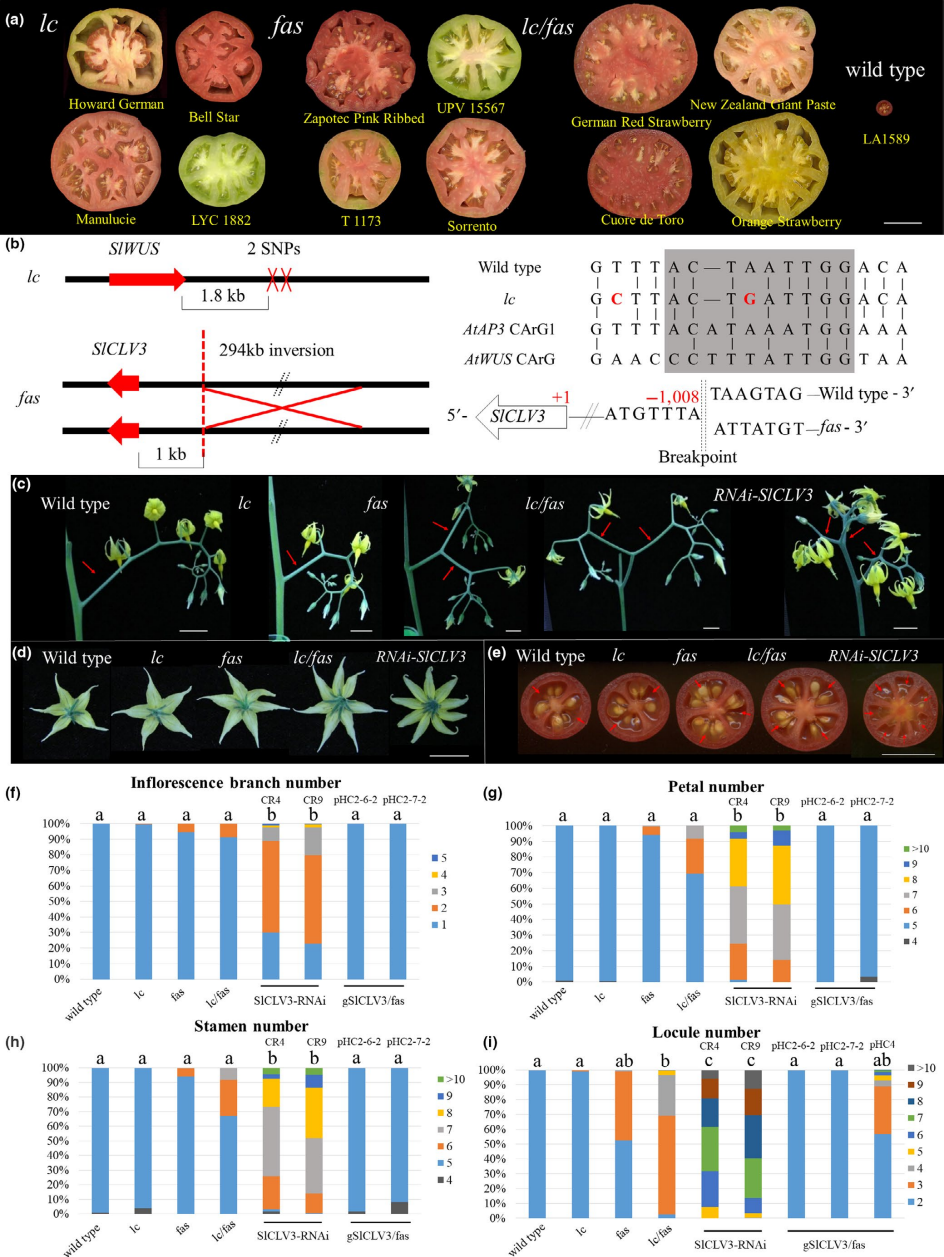
The effect of natural *lc* and *fas* mutant alleles on floral organs and inflorescence development. (a) Tomato varieties containing *lc* and/or *fas* mutant alleles carry multilocular fruits. The wild type tomato (*Solanum pimpinellifolium* LA1589) typically contains only two locules. Size bar = 3 cm. (b) Genomic sequence changes in *lc* and *fas*. The genomic sequence underlying the *lc* mutation shares similarity to the CArG box of Arabidopsis *AP3*. The two SNPs underlying *lc* are marked in red and the putative CArG box is highlighted in gray. The *fas* mutation is caused by a ~294 kb inversion with a breakpoint in the promoter region of *SlCLV3*. (c–e) Inflorescences, flowers and fruits of *lc*,* fas*,* lc/fas* NILs and *SlCLV3*‐RNAi lines. Bar = 1 cm. (f–i) The ratio of branched inflorescences and floral organ number in NILs and various transgenic lines. RNAi‐CR4 and RNAi‐CR9 represent two independent *SlCLV3*‐RNAi transgenic lines. pHC2‐6‐2 and pHC2‐7‐2 represent two independent transgenic lines that were transformed with *SlCLV3* genomic sequence driven by a 5.5 kb promoter construct. pHC4 contained a shorter *SlCLV3* promoter that served as a negative control for the complementation test. Pairwise comparisons were made between the genotypes using ANOVA and means were separated with Tukey's HSD test with *p* < 0.05

The natural mutations at the *lc* and *fas* loci underlie the orthologs of the Arabidopsis meristem organization genes *WUS* and *CLV3*, and were originally identified due to their strong effects on locule number (Figure 1a) (Barrero, Cong, Wu, & Tanksley, 2006; Lippman & Tanksley, 2001; Muños et al., 2011; Rodríguez et al., 2011; Xu et al., 2015). The associated nucleotide polymorphisms are 3′ of *SlWUS* (Muños et al., 2011; van der Knaap et al., 2014) and the promoter region of *SlCLV3* (Huang & van der Knaap, 2011; Xu et al., 2015), respectively (Figure 1b). The *lc* mutations may correspond to the CArG box, which is critical to suppress *WUS* expression in Arabidopsis (Liu et al., 2011). We determined the effects of *lc* and *fas* on fruit morphology and reproductive traits by creating near‐isogenic lines (NILs) in the wild species *Solanum pimpinellifolium* accession LA1589 background that differed only for the alleles at these two loci. In addition to more locules, the expectation is that these meristem organization mutants would lead to increased inflorescence branching (Park, Jiang, Schatz, & Lippman, 2012). Wild type tomato typically develop a single‐branch inflorescence with a reiterating pattern of an IM terminating into an FM and the formation of a new IM along the flank of the FM (Figure S1b), whereas *fas* and especially *lc/fas* NILs show a significant increase in inflorescence branching (Figure 1c,f). For example, the first inflorescence in the *fas* and *lc/fas* NILs nearly always forms a branched architecture — as two IMs appear to emerge from a single terminating SAM (Figure S2). *lc*/*fas* also resulted in the highest floral organ number among the NILs especially for locule number (Figure 1d–e,g–i). Overall, *lc* alone had no effect on inflorescence branching and floral organ number unless in combination with *fas* (Table S1). Regardless, the most dramatic floral organ number change was for locule number in the natural lc and fas mutants.

**Figure 2:**
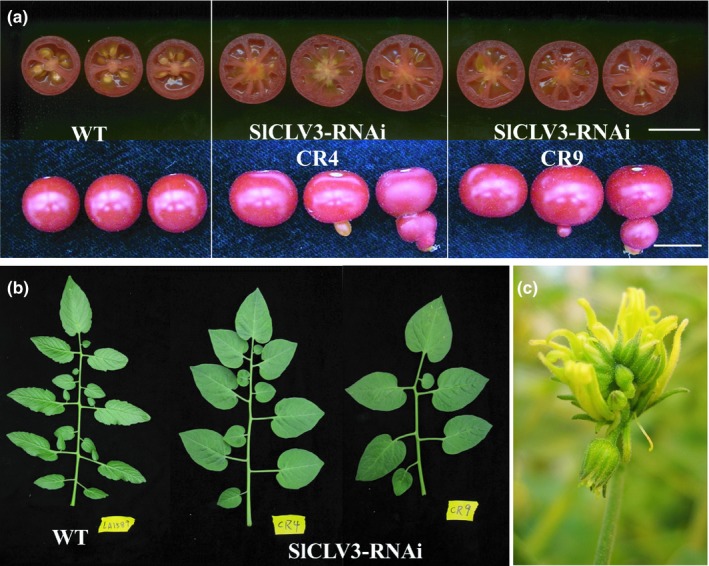
Phenotypic analysis of *SlCLV3*‐RNAi plants. (a) Cut fruits of wild type and *SlCLV3*‐RNAi lines. The fruits show aberrant seed development and ectopic fruit structure, with extra carpels produced inside the primary carpel. Size bar = 1 cm. (b) Images of leaves of wild type and *SlCLV3*‐RNAi plants. (c) Formation of an ectopic inflorescence inside a flower in *SlCLV3*‐RNAi plants

*SlCLV3* has been demonstrated to underlie *fas* (Xu et al., 2015), which implies that the *fas* inversion has compromised the promoter of this gene. We also downregulated *SlCLV3* expression by expressing part of the coding region as an RNAi construct in stably transformed tomato. As expected, the tomato plants transformed with the pHC2 construct, which contained approximately 5 kb of the wild type promoter and the entire coding region of the gene, rescued the bi‐locular fruit phenotype in the *fas* background, whereas the shorter promoter construct pHC4 did not (Figure 1i). On the other hand, downregulation of *SlCLV3* led to an increase in all floral organs as well as inflorescence branching (Figure 1c–i). Additional phenotypes associated with the severely reduced expression of *SlCLV3* included nearly seedless fruits, indeterminate meristematic activity as evidenced by the development of ovaries within the initial ovary, widened leaflets with smooth margins, decreased number of secondary leaflets, and reduced complexity of the compound leaf (Figure 2a–b). Extreme phenotypes in the flowers were also occasionally observed, such as an inflorescence that was reinitiated inside a flower (Figure 2c).

To determine whether the selection of *lc* and *fas* during domestication might have been due to the associated increases in fruit weight, we evaluated the effect of the natural mutations on this trait. In the wild species background, fruit weight was only significantly increased in the *lc/fas* double NIL (Table 1a). Fruit area was significantly increased in *fas* and even more in the *lc/fas* double NIL. This suggested that the fruits were wider but flatter and thus the increase in locule number did not yield much heavier fruits in the LA1589 background. To determine whether *lc* and *fas* exerted a synergistic effect on locule number, we evaluated the genetic effect between these two loci (Table 1b). The epistatic analysis was performed using two‐way ANOVA and showed a significant interaction between *lc* and *fas* for locule number (*p*‐value < 0.001) but not for other traits (Table 1b). The degree of dominance of each locus showed that *lc* affected locule number in a mostly additive manner. On the other hand, the *fas* mutation was nearly completely recessive over the wild type with a d/a value of −0.88 (Table S2).

**Table 1:** The effects of *lc* and *fas* mutations on fruit size, weight and locule number

	Plant N	Fruit perimeter (cm)	Fruit area (cm^2^)	Fruit weight (g/per fruit)
(a)				
Wild type	6	3.707 ± 0.083 a	1.000 ± 0.043 a	0.810 ± 0.061 a
*lc*	5	3.773 ± 0.106 a	1.034 ± 0.056 a	0.835 ± 0.063 a
*fas*	5	3.943 ± 0.094 b	1.125 ± 0.054 b	0.914 ± 0.063 ab
*lc/fas*	6	4.095 ± 0.092 b	1.215 ± 0.055 c	0.971 ± 0.060 b

Population	Traits	*p*‐value		
		*lc*	*fas*	*lc x fas*
				(b)
12S190(BC_8_)	Locule number	<0.001	<0.001	<0.001
	Fruit weight	<0.001	<0.001	0.963
13S133(BC_9_F_2_)	Locule number	<0.001	<0.001	<0.001
	Fruit weight	0.063	<0.001	0.544
	Fruit area	0.003	<0.001	0.234

(a) Comparisons of fruit perimeter, area and weight between the wild type, *lc, fas* and *lc/fas* NILs. The fruit weight represents the average value of 20 ripe fruits from 5 to 6 individual plants per genotype. The average fruit perimeter and area were measured from 8 to 10 ripe fruits. Pairwise comparisons between the NILs were performed using ANOVA and means were separated using Tukey's HSD test. (b) Effects and interactions of *lc* and *fas* on the traits of mature fruits. Significant effects and interactions were shown by the *p*‐values computed from the F ratio in ANOVA.

### 2.2 *lc* and *fas* produce fasciated inflorescences and enlarged meristems

**Figure 3:**
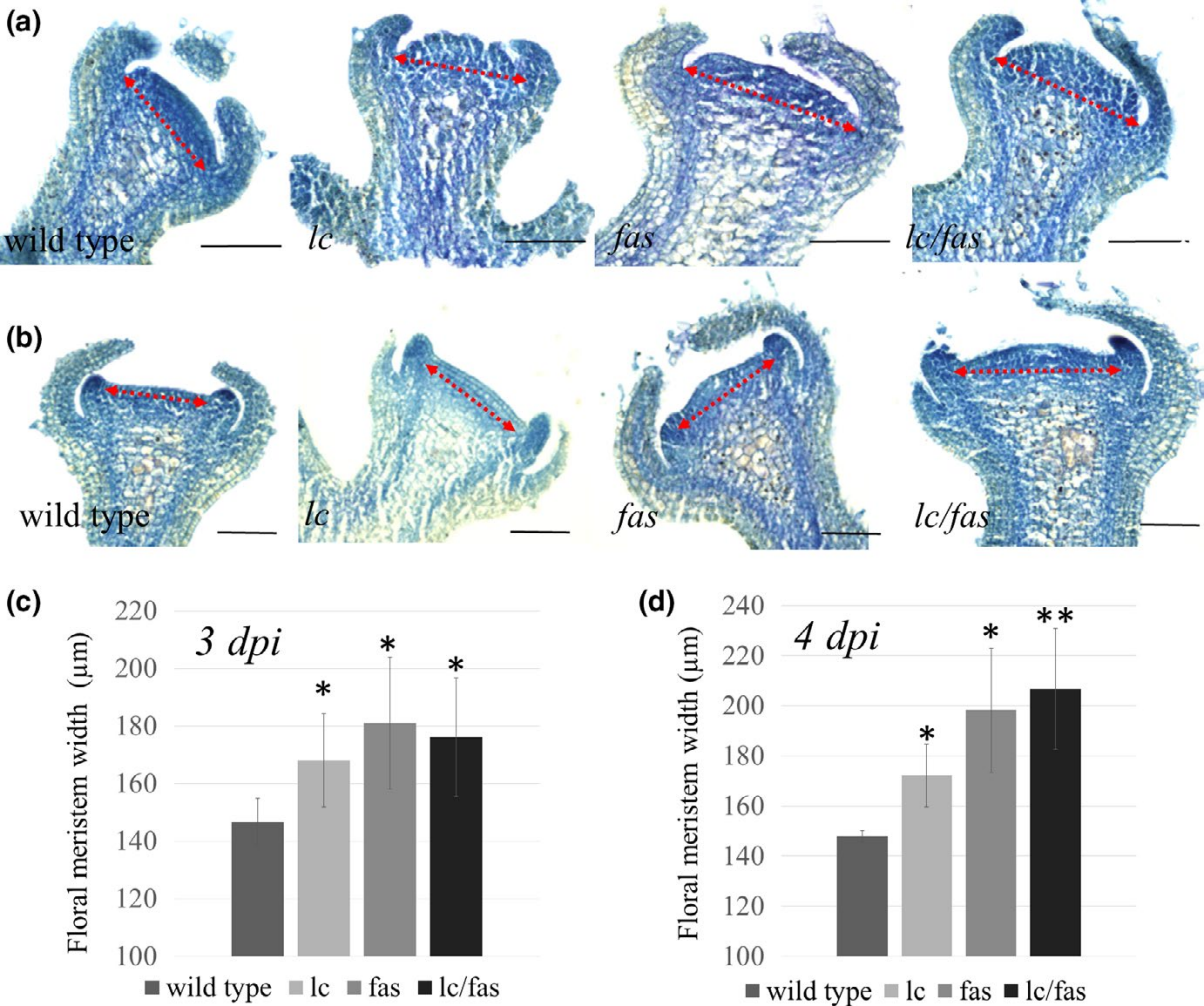
Floral meristem enlargement and fasciated inflorescences caused by *lc* and *fas*. (a–b) Longitudinal section of floral meristem of *lc*,* fas*,* lc*/*fas* NILs and the wild type at 3 and 4 dpi. The red dash arrow marks the width of each meristem. (c–d) Floral meristem width measured in 5–9 buds. Error bar denotes the standard deviation. A two‐tailed *t*‐test was performed between mutants and the wild type. Significant differences are indicated by asterisks. *p < 0.01, **p < 0.001. Scale bar = 100 μm

To determine whether higher locule number was associated with increased size of the floral meristem, we compared their widths in young floral buds prior to the emergence of the carpel primordia. At 3 days post floral initiation (dpi), when the floral meristem was not yet enclosed by sepal primordia, FMs of single and double mutants were significantly wider than that in the wild type (Figure 3a,c). At 4 dpi after the initiation of petal primordia, the FM enlargement in *fas* was even more pronounced than at 3 dpi, especially in the *lc/fas* double mutant background (Figure 3b,d). For example, while wild type meristem stayed around the same width of approximately 147 μm, the *lc/fas* double NIL increased from 176 to 207 μm from 3 to 4 dpi. Notably, the synergistic effect between *lc* and *fas* on meristem size was not observed in floral buds at 3–4 dpi, suggesting that the interaction might happen at a later developmental stage. The data showed a positive trend between the floral meristem size and locule number resulting from the effects of *lc* and *fas*. In addition, *fas* allowed an extended period of meristem expansion compared to the wild type and *lc*.

### 2.3 *lc* and *fas* affect the expression of *SlCLV3*,* SlWUS*,* SlYABBY2*, and *TAG1*

**Figure 4:**
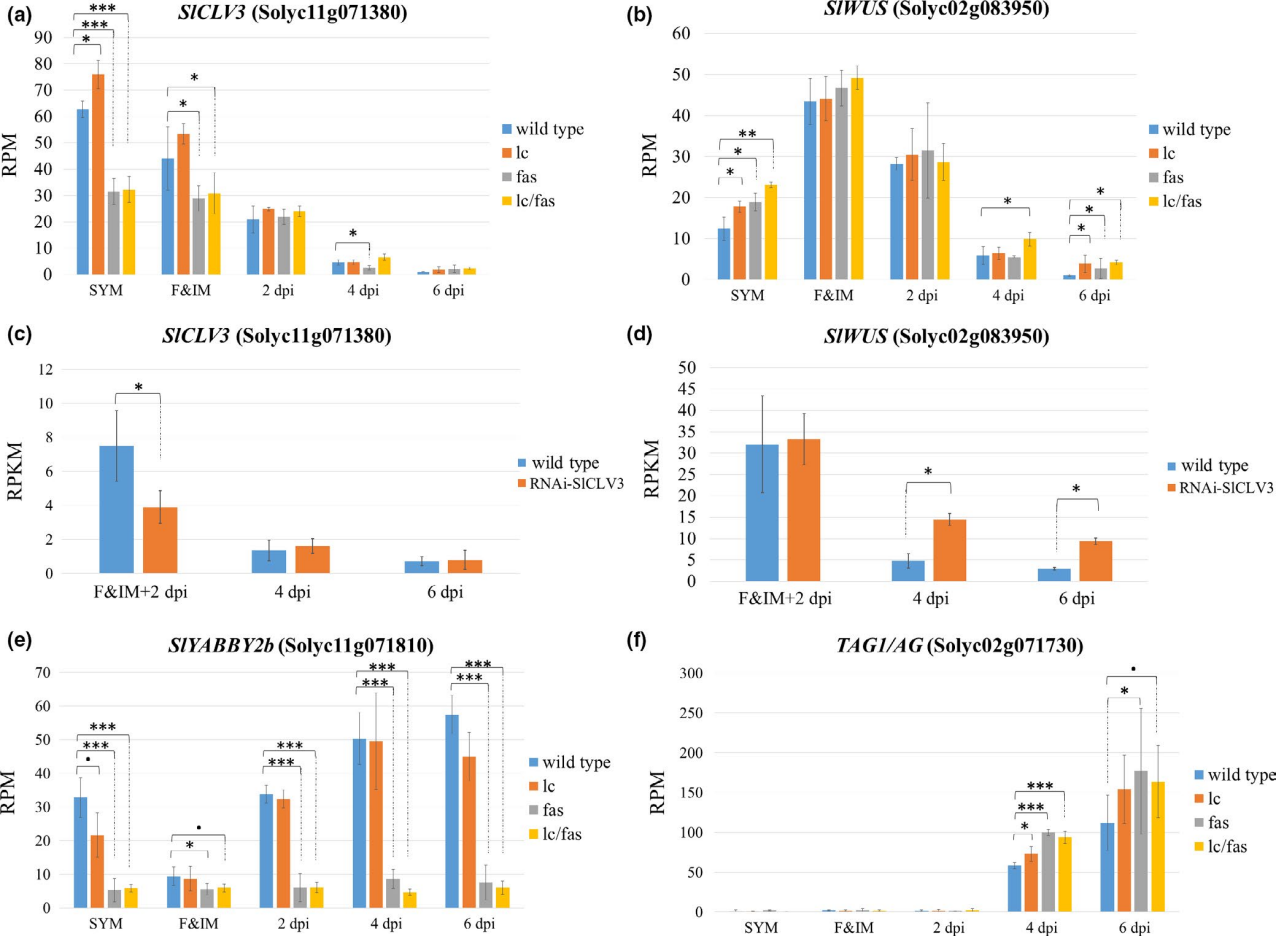
RNA‐seq analysis of *SlCLV3*,* SlWUS*,* SlYABBY2* and *TAG1* during floral development. Tissues were collected from *lc*,* fas*,* lc*/*fas*, RNAi‐*SlCLV3*, and the wild type plants at five developmental stages: sympodial shoot apical meristem (SYM), floral meristem with inflorescence meristem (F&IM), 2, 4 and 6 dpi. Shown are the normalized expression of *SlCLV3* (a, c) and *SlWUS* (b, d)*, SlYABBY2b* (e) and *TAG1* (f). The expression levels obtained from 3′ Tag RNA‐seq method (a, b, e, f) were normalized using reads per million reads (RPM), while data obtained from whole mRNA‐seq method (c, d) were normalized using reads per kilobase million reads (RPKM). The *p*‐value was obtained from linear‐based likelihood ratio test between mutants and the wild type using DEseq2 in R. Data are shown as means ± *SD* from three to four biological replicates. Significant differences are represented by asterisks. •*p* < 0.1, *p < 0.05, **p < 0.001 and ***p < 0.0001 (a, b, e, f). * adjusted *p* < 0.05 (c, d)

To reveal the effect of the *lc* and *fas* mutations on gene expression, we performed expression analyses using meristems and young floral buds from the wild type and mutant NILs. RNA was isolated from sympodial shoot apical meristem (SYM), the combined floral and inflorescence meristem (F&IM), and floral buds at 2, 4, and 6 dpi (Figure S1b). As expected, the expression of *SlCLV3* was significantly lower in the SYMs and F&IMs in lines carrying *fas* compared to that in the wild type (Figure 4a). On the other hand, a significant increase in *SlCLV3* expression, specifically in SYMs, was detected in *lc*. This supported the notion that the two SNPs at CArG box in *lc* (Figure 1b) abolish the binding of a suppressor, resulting in increased expression of *SlWUS* and *SlCLV3* in SYMs. After 2 dpi, expression of *SlCLV3* was similar among the genotypes. *SlWUS* expression was significantly increased in the single and double mutants in the SYM but not in the F&IM. At the relatively late developmental stage of 6 dpi, *SlWUS* expression was higher in all mutants implying a delayed termination of gene expression (Figure 4b).

A significant reduction in *SlCLV3* expression resulting from the *SlCLV3* RNAi construct was also found in F&IM and 2 dpi floral bud tissues compared to the wild (Figure 4c). The residual *SlCLV3* expression was likely due to incomplete RNAi or the transcripts derived from the hairpin RNAi construct. Severe downregulation of *SlCLV3* also led to higher expression of *SlWUS* but only at 4 and 6 dpi (Figure 4d). Together, these results showed that the reduction in *SlCLV3* expression could lead to a delay in the suppression of *SlWUS*.

The genomic inversion in *fas* also resulted in a breakpoint in the first intron of *SlYABBY2b* (Figure 1b). As a result, *SlYABBY2b* expression persisted at low levels in *fas* and *lc/fas* at all stages, suggesting that the mutation did not fully block *SlYABBY2b* transcription (Figure 4e). However, almost all detected transcripts mapped to the 5′ region of *SlYABBY2b* comprising the first exon and before the breakpoint of the *fas* inversion (Figure S3). This indicated that the mutation led to the production of truncated RNAs that are likely non‐functional.

We wanted to evaluate whether the *WUS*‐dependent *AG* expression found in Arabidopsis is conserved in tomato. The expression of tomato *AG* homolog *TAG1* was significantly increased in *lc*,* fas*,* lc/fas* floral buds at stage 4 dpi (Figure 4f). These differences were also observed in mutants at 6 dpi albeit to a lesser extent than at 4 dpi. Notably, the synergistic effect of *lc* and *fas* was not observed from *TAG1* expression, suggesting that *SlWUS* solely controls *TAG1* expression. It is conceivable that TAG1 leads to downregulation of *SlWUS* though its binding to the CArG box located 3′ of *SlWUS*. However, the increased expression of *TAG1* in *lc*,* fas* and *lc*/*fas* NILs was not associated with reduced *SlWUS* expression suggesting that *TAG1* might not directly involved in *SlWUS* repression.

### 2.4 Pronounced temporal‐spatial expression changes of *SlCLV3* and *SlWUS* in the *lc* and *fas* NILs

**Figure 5:**
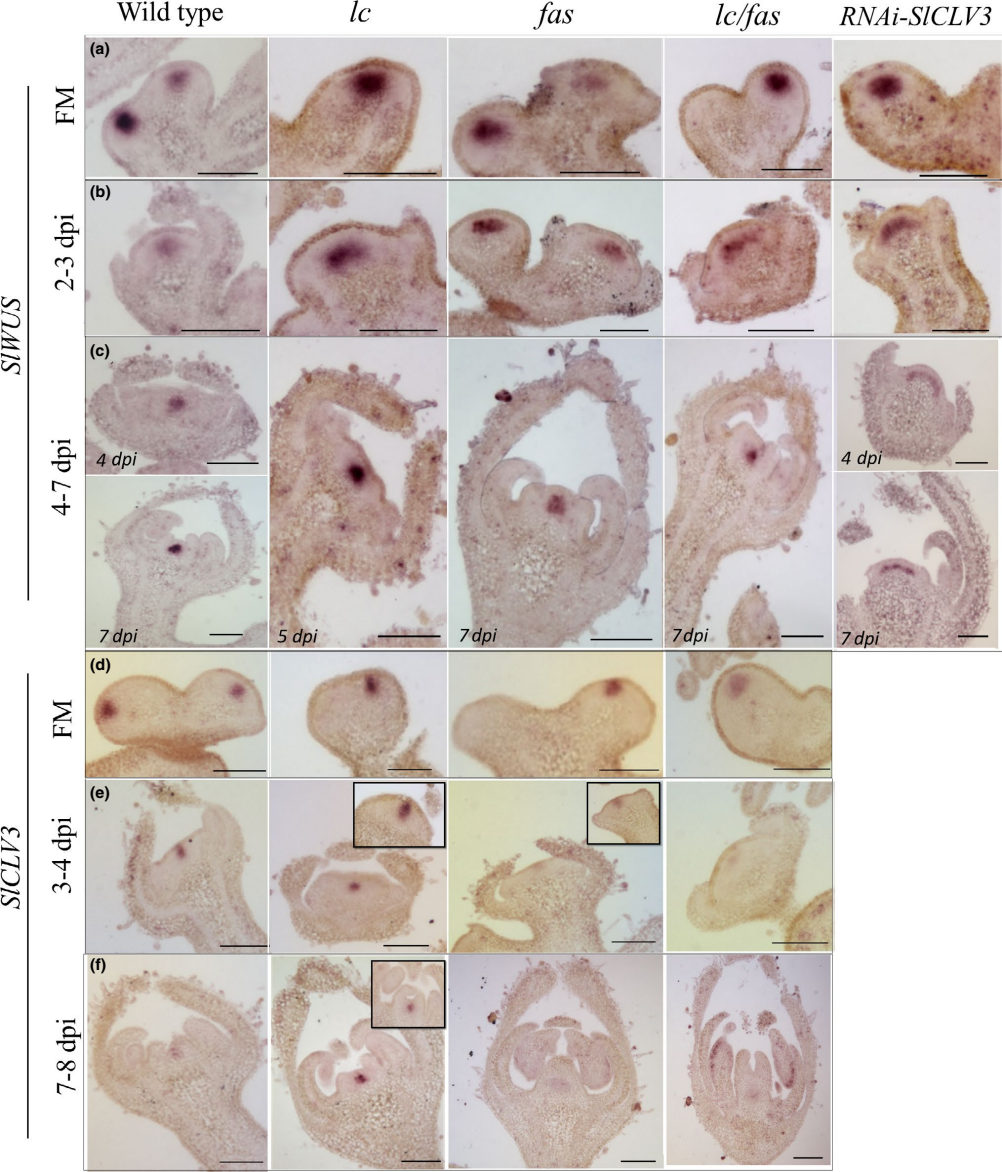
Expression domains of *SlWUS* and *SlCV3* in tomato floral meristems. (a–c) *SlWUS* expression domain in wild type, *lc*,* fas*,* lc*/*fas* NILs and RNAi‐*SlCLV3* lines in floral meristems, floral buds at 2–3 dpi with emerged sepal primordia, floral buds at 4 dpi with emerged petal primordia, and floral buds at 7 dpi which carpel primordia formed a central column. (d–f) *SlCLV3* expression domain in wild type, *lc*,* fas*, and *lc*/*fas* NILs in floral meristems, floral buds at 3–4 dpi and floral buds at 7–8 dpi. The figure inserts show additional tissue sections of the same genotype at the same developmental stages. The genes used as probes are shown on the left. Scale bar = 100 μm

To determine whether temporal‐spatial expression patterns of *SlCLV3* and *SlWUS* were altered in the NILs, we performed RNA in situ hybridization (Figure 5). In FM, the spatial expression of *SlWUS* revealed no dramatic changes in expression level among the genotypes (Figure 5a), a finding that was consistent with results from the RNA‐seq analyses (Figure 4). At 2–3 dpi, we observed an expansion of the *SlWUS* expression domains in the single and double NILs compared to that in the wild type (Figure 5b). At 4–7 dpi, *SlWUS* expression in the NILs was constrained again to the center and appeared similar to the pattern found in the wild type (Figure 5c). The most substantial change in *SlWUS* expression pattern was found in the *SlCLV3‐*RNAi lines (Figure 5a–c). Contrary to the single and double NILs, the *SlCLV3*‐RNAi led to a lateral expansion of the *SlWUS* expression domain in the floral buds especially at the later stages of bud development.

Expression of *SlCLV3* in the FM showed a similar pattern as *SlWUS* (Figure 5d). While *SlCLV3* exhibited a similar expression pattern between *lc* and the wild type in FMs and 3–4 dpi tissues, it is expressed at a much higher level in *lc* at 7–8 dpi (Figure 5d–f). By contrast, the *fas* and *lc/fas* mutants led to a weaker expression of *SlCLV3* in the floral buds at 3–4 or 7–8 dpi (Figure 5d–f). These RNA *in situ* hybridization results are complementary with the RNA‐seq results in supporting the expression changes of *SlCLV3* and *SlWUS* in NILs and RNAi plants.

### 2.5 Differentially expressed genes in *lc* and *fas*

**Figure 6:**
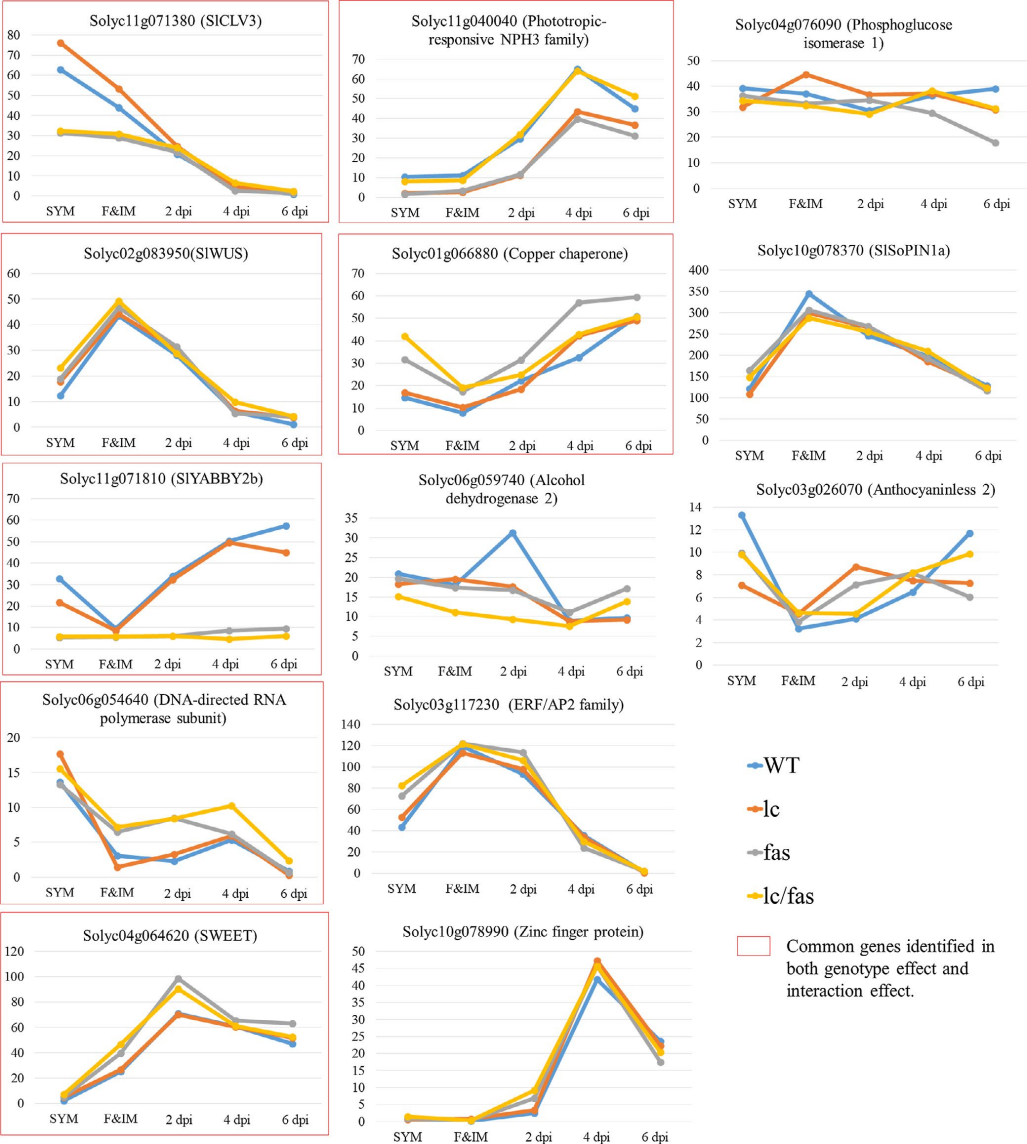
Differentially expressed genes with significant genotype × development (G × D) interaction effects. *Y*‐axis represents the RPM value. *X*‐axis represents five different developmental stages

To decipher the genome‐wide gene expression changes resulting from mutations in *lc* and *fas*, we applied linear factorial analysis on the RNA‐seq results across the five developmental stages. Differentially expressed genes (DEGs) that were consistently up‐ or down‐regulated across different stages were identified as genes with significant genotype effect, while DEGs responding differently to genotypic and developmental variations (ex: showing dynamic expression patterns across stages) were considered as genes with significant genotype by developmental (G × D) effects. A total of 669 and 13 DEGs were identified with significant genotype (Padj < 0.1) and G × D interaction effects (*p* < 0.001), respectively (Data S1), which is a relatively low threshold level. The low number of genes that were identified under the model implied that very few differentially expressed genes resulted from one mutation or the other. This finding is consistent with the notion that *lc* and *fas* are not null mutations, and therefore have a relatively weak impact on downstream genes of either mutation. With seven genes shared by the two categories (669 DEGs in the “genotype effect” and 13 DEGs in the “G × D effect”), a total of 675 unique DEGs were used for the downstream data analyses. The 13 DEGs that showed G × D interaction included the known *SlCLV3* (*Solyc11 g071380*), *SlWUS* (*Solyc02 g083950*) and *SlYABBY2b* (*Solyc11 g071810*) (Figure 6). Genes involved in nutrient and hormone transport included the sugar transporter *SlSWEET1b* (*Solyc04 g064620*) and the auxin efflux carrier *SlSoPIN1a* (*Solyc10 g078370*). The *SlSWEET1b* is a homolog of Arabidopsis *AtSWEET1* that encodes a transmembrane sugar transporter (Chen et al., 2010; Feng, Han, Han, & Jiang, 2015). Sugar transporters are known to affect meristem development by regulating sugar accumulation and distribution in the meristem (Francis & Halford, 2006; Lastdrager, Hanson, & Smeekens, 2014). The *slpin1a* loss‐of‐function mutation, also known as *entire‐2*, causes aberrant organ positioning in the shoot, inflorescence and floral meristems by disrupting directional auxin transport (Martinez, Koenig, Chitwood, & Sinha, 2016). We also identified genes encoding metabolic enzymes such as glucose‐6‐phosphate isomerase (*Solyc04 g076090*), *ALCOHOL DEHYDROGENASE 2*/*SlADH2* (*Solyc06 g059740*) involved in fatty acid degradation and the production of volatiles during fruit ripening (Speirs et al., 1998), and a close homolog of Arabidopsis *ANTHOCYANINLESS 2* (*ANL2*) in Arabidopsis (*Solyc03 g026070*), which affects anthocyanin accumulation (Kubo, Peeters, Aarts, Pereira, & Koornneef, 1999). DEGs with G × D interaction effects also included a NON‐PHOTOTROPIC HYPOCOTYL 3 (NPH3) protein (*Solyc11 g040040*) that is involved in phototropic responses and protein ubiquitination in Arabidopsis (Gingerich et al., 2005; Pedmale & Liscum, 2007).

### 2.6 Cluster analysis and DEGs co‐expressed with *SlWUS*

**Figure 7:**
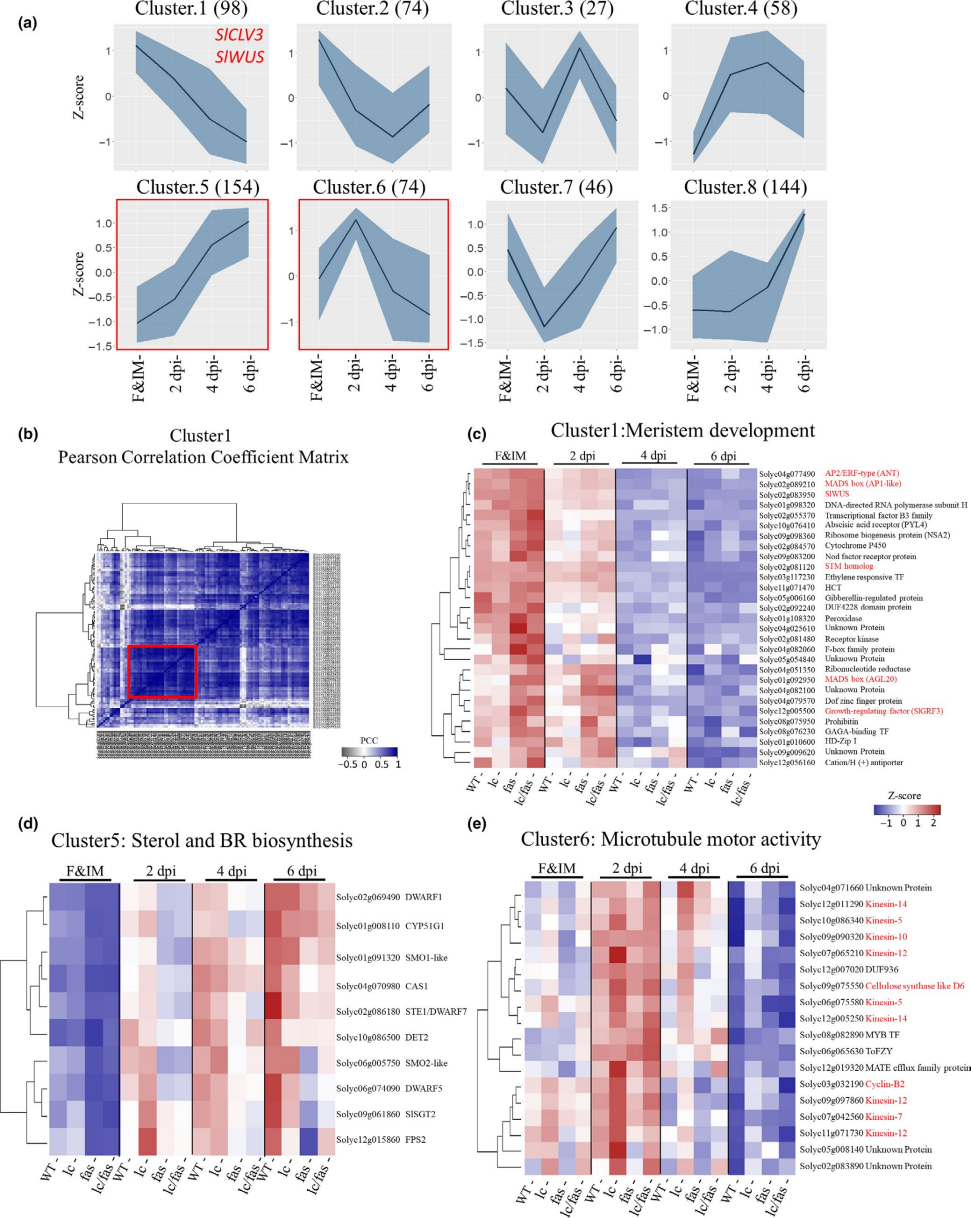
Expression profiles of the co‐expressed gene clusters. (a) Eight co‐expressed clusters identified from normalized expression values (z‐scores) of the wild type samples are clustered using K‐mean algorithm in Mfuzz (R package). The dark blue lines represent the average of expression values, whereas the light blue regions represent the maximum and minimum expression values. (b) Pearson Correlation Coefficient matrix based on the expression of genes in WT and mutants in cluster 1. The core genes co‐expressed with *SlWUS* are marked by the red square. (c) Heatmap of core genes co‐expressed with *SlWUS* in cluster 1. Normalized expression values were used for hierarchical clustering. (d) Cluster 5 enriched with genes involved in sterol biosynthesis. Although *Solyc10 g086500* was not significantly differentially expressed below the adj. *p* < 0.1 threshold, it was included in this analysis based on *p*‐value < 0.01 and its GO signature. (e) Cluster 6 enriched with genes involved in microtubule motor activity and cell cycle processes in cluster 6. Genes highlighted in red are putatively involved in microtubule binding activity and cytokinesis

The linear factorial modeling identified a large collection of DEGs that were consistently up‐ or down‐regulated in all developmental stages as well as those showing genotype‐ and developmental stage‐ dependent differential expressions. To identify groups of DEGs with similar expression dynamics among the 675 DEGs dataset, we conducted fuzzy C‐means clustering using corresponding genes with normalized expression levels in the WT. This led to the identification of eight co‐expressed clusters with cluster 1 representing *SlWUS* and *SlCLV3* (Figure 7a). Clusters with DEGs that showed higher expression at early developmental stages (cluster 1, 2, 6) were highly enriched in GO terms for “stem cell population maintenance” and “microtubule motor activity” (Table 2). DEGs with higher expression at the later developmental stages (cluster 5 and 8) were specifically enriched in “steroid biosynthetic processes”. Cluster 1, 5 and 6 were selected as they contained the GO terms that were most significantly overrepresented in any of the clusters (adj. *p*‐value < 1e‐05).

**Table 2:** Enriched GO terms in each co‐expressed gene cluster

	GO term	adjP(BH)	#G	Arabidopsis Homolog
Cluster1	Stem cell population maintenance	1.90E‐06	6	[AGL20, ANT, CLV3(SlCLE15/FAS), LHW, STM, WUS]
	Reproductive structure development	3.40E‐03	11	[ACX4, AGL20, ANT, AP1, APX1, FES1, PIN1, SMT1, SPL15, STM, WUS]
	Oxidoreductase activity	5.80E‐03	3	[ALDH10A8, ALDH22A1, CER4]
	Response to reactive oxygen species	7.40E‐03	4	[APX1, CCS, CSD2, CYT1]
	Establishment of protein localization to organelle	3.20E‐02	3	[ACX4, AGL20, ATERDJ2A]
	Amine metabolic process	3.60E‐02	3	[ALDH10A8, GDU1, STM]
	Cytoskeleton organization	4.30E‐02	3	[EHD2, PLE, RPL3B]
Cluster2	Anatomical structure morphogenesis	3.20E‐02	6	[CLE41(SlCLE13), CLV3(SlCLE9), DFL1, GID1C, LOX1, UGT74E2]
	Post‐embryonic organ development	4.10E‐02	3	[ARPN, GID1C, LOX1]
	Cellular response to lipid	4.60E‐02	3	[AREB3, GID1C, UGT74E2]
	Monocarboxylic acid metabolic process	2.40E‐02	5	[GAPC2, LACS4, LOX1, MOD1, UGT74E2]
Cluster3	NA			
Cluster4	Phenylpropanoid metabolic process	8.70E‐03	3	[ATR2, PAL2, UGT72E1]
	Transferase activity, transferring acyl groups	3.20E‐02	3	[ACLA‐2, ASAT1, ICL]
	Tissue development	2.70E‐02	4	[ETC1, PIR121, YA]
Cluster5	Oxidoreductase activity	2.40E‐03	5	[ABA2, CAD9, DWF1, HPR, XDH1]
	Response to karrikin	4.00E‐04	5	[ELF4, GI, PAL1, UGT78D2, ZIFL1]
	Phenylpropanoid metabolic process	3.90E‐04	6	[4CL3, APRR2, CAD9, DWF1, PAL1, UGT78D2]
	Pollen development	1.00E‐02	5	[4CL3, BT1, CAS1, PAL1, UTR3]
	Homeostatic process	2.30E‐02	6	[CAX3, CRY2, HA1, IAMT1, NHX2, WCRKC1]
	Response to oxidative stress	2.10E‐04	9	[BT1, CYT1, GI, HSFA2, LOL1, OXS3, PAL1, WCRKC1, XDH1]
	Response to water deprivation	3.90E‐04	8	[ABA2, CRY2, HA1, HPR, PAL1, SIP3, XDH1, ZIFL1]
	Regulation of reproductive process	1.00E‐02	5	[CAL, CRY2, ELF3, ELF4, GI]
	Response to light stimulus	1.20E‐02	9	[ASN1, CRY2, ELF3, ELF4, GI, HPR, HSFA2, JAC1, PAL1]
	Steroid biosynthetic process	1.40E‐06	7	[CYP51G1, DWF1, DWF5, FPS2, SMO1‐1, SMO2‐1, STE1]
Cluster6	Regionalization	3.50E‐04	4	[AN3, KAN, PHB, SCR]
	Microtubule motor activity	8.10E‐06	4	[AT1G72250, ATK1, TES, ZWI]
Cluster7	Fruit development	3.20E‐02	4	[CRF2, DCP2, EFE, USPL1]
	Single‐organism catabolic process	3.20E‐02	4	[CYSC1, GSTF8, PGI1, PLP4]
	Shoot system development	1.50E‐02	6	[CRF2, CT‐BMY, DCP2, FLA1, LBD37, PGI1]
	Response to cytokinin	8.60E‐04	5	[AHP1, APA1, CRF2, HAT22, ZFP6]
Cluster8	Response to inorganic substance	4.30E‐04	14	[AT4G39130, CCH, ECP63, EIN3, ERD10, GAD, HB‐7, LEA4‐5, LTP2, OASA1, OXS3, PDR12, RD26, SOX]
	Response to acid chemical	2.00E‐02	13	[AFP1, AT4G39130, ECP63, ERD10, GPCR, HB‐7, LEA4‐5, LTP2, MYB48, PDR12, RD26, SCL14, TT4]

*p*‐value was adjusted using the Benjaminin‐Hochberg (BH) correction.

Cluster 1 was strongly enriched with genes involved in stem cell population maintenance, consistent with the notion that their expression patterns were negatively correlated with the developmental gradient. Genes enriched for meristem maintenance included *SlCLV3*,* SlWUS*, putative homologs of the Arabidopsis *SHOOT MERISTEMLESS* (*STM*) (*Solyc02 g081120*), *AGAMOUS‐like 20* (*AGL20*) (*Solyc01 g092950*), *AINTEGUMENTA* (*ANT*) (*Solyc04 g077490*) and *LONESOME HIGHWAY* (*LHW*) (*Solyc06 g074110*).

To identify genes that might work in concert with *SlWUS*, the Pearson's correlation coefficients (PCCs) between gene pairs in cluster 1 were calculated using the normalized expression levels from mutants and WT. As shown in Figure 7b,c, a set of genes were identified with a high correlation to *SlWUS* expression. The subset of 29 genes was highly expressed in the F&IM, and then their expression diminished during the termination of the meristematic potential of the remaining FMs as the floral organ primordia arose (Figure 7c). These genes showed higher expression in *fas* and *lc*/*fas* compared to WT, suggesting that the upregulation might be related to the expansion of *SlWUS* expression domain (Figure 5). The identification of co‐expressed tomato *SHOOT MERISTEMLESS* (*STM*) with *SlWUS* suggested that some genes in this cluster might be related to stem cell function. Indeed, six of the 29 genes highlighted in red (Figure 7c) are involved in meristem or floral development based on previous studies in Arabidopsis (Irish & Sussex, 1990; Krizek, 2011; Lee et al., 2000; Lenhard, Jürgens, & Laux, 2002; Omidbakhshfard, Proost, Fujikura, & Mueller‐Roeber, 2015).

Cluster 5 was enriched with genes encoding enzymes in sterol biosynthesis. They included putative orthologs of *DWARF1* (*Solyc02 g069490*), *DWARF5* (*Solyc06 g074090*), *DWARF7* (*STE1*) (*Solyc02 g086180*) and *DET2* (*Solyc10 g086500*) (Figure 7d). In contrast to genes co‐expressed with *SlWUS,* the expression of the sterol biosynthesis pathway genes increased with the floral development stages. In addition, these genes were expressed at lower levels in *fas* and *lc*/*fas* compared to WT across different developmental stages, implying the negative roles of these genes in meristem maintenance. As sterols are precursors of brassinosteroids (BRs), membrane components, and signaling molecules during plant development (Vriet, Russinova, & Reuzeau, 2013), these results have provided new insights into the potential roles of sterols/BRs in FM regulation.

Cluster 6 was enriched with genes encoding microtubule motor proteins, including putative homologs of Arabidopsis *KINESIN 1* (*ATK1*) (*Solyc12 g005250*), *PHRAGMOPLAST‐ASSOCIATED KINESIN‐RELATED PROTEIN 1* (*PAKRP1)* (*Solyc09 g097860*), *TETRASPORE* (*TES*) (*Solyc07 g042560*) and *ZWICHEL* (*ZWI*) (*Solyc12 g082730*). These genes were expressed at higher levels at 2 dpi, indicating that their expression might be positively regulated by genes mediating outer whorl initiation. Phylogenetic analysis revealed that these kinesin genes belonged to different subfamilies (Figure S4), and the co‐expression signature of these genes suggested that they might act cooperatively in cell division and cell growth during floral development. In addition to the genes encoding kinesin proteins, the genes closely related to *Cyclin B2;3* (*Solyc03 g032190*) and *Cellulose Synthase‐Like D5* (*Solyc09 g075550*) were also found in cluster 6. Although kinesins might also play a role in organelle movement (Zhu & Dixit, 2012), the co‐expressed pattern of these kinesin genes with putative tomato *Cyclin B2;3* and *Cellulose Synthase‐Like* gene suggested that they were more likely involved in cytokinesis (Hunter et al., 2012; Tank & Thaker, 2011).

## 3 DISCUSSION

### 3.1 Conserved regulatory mechanism between LC‐FAS and WUS‐CLV3

In the recent years, a number of genes associated with the control of tomato fruit shape and size have been identified (Mu et al., 2017; van der Knaap & Østergaard, 2017; van der Knaap et al., 2014). The key genes that contribute to the flat shape and multi‐loculed tomato fruits are *SlWUS* and *SlCLV3* (Muños et al., 2011; Xu et al., 2015). The origin and distribution of the mutant alleles of these genes, including their effect on tomato fruit morphology was investigated at the population level (Blanca et al., 2015; Rodríguez et al., 2011). While *lc* appears to have arisen in wild relatives, *fas* arose later during domestication (Blanca et al., 2015). Although *fas* appears to be more effective in producing fasciated fruits than *lc* (Rodríguez et al., 2011), it is not well understood how each locus alone or in combination controls fruit development and gene expression due to differences in the genetic background of the tomato accessions studied.

Our findings indicate a close conservation between tomato LC‐FAS and Arabidopsis WUS‐CLV3 regulatory loops. Results of expression analysis revealed that *SlCLV3* was significantly downregulated and *SlWUS* was significantly upregulated in the SYM of *fas*. On the other hand, as *SlWUS* was upregulated in *lc*,* SlCLV3* was accordingly upregulated in SYM in this mutant background. These results support our hypothesis that *lc* is caused by a gain‐of‐function mutation of *SlWUS*, while *fas* is caused by a partial loss‐of‐function mutation of *SlCLV3*. Although the LC‐FAS negative feedback regulatory loop was conserved in the SYMs, the lower expression of *SlCLV3* in the FM of *fas, lc/fas* and RNAi‐*SlCLV3* did not result in an increase of *SlWUS* expression in F&IM (Figure 6). This raised a possibility that other factors could compensate the effect of *SlCLV3* and maintain the suppression of *SlWUS* in FM. Interestingly, the *SlCLE9* gene, a member of the *CLV3/EMBRYO‐SURROUNDING REGION* (*CLE*) gene family, was identified as one of the top candidates that showed a significant genotypic effect (Figure S5). *SlCLE9* expression was five‐fold higher in the F&IMs of *fas* and *lc*/*fas* NILs as compared to the wild type, while being ten‐fold higher in RNAi‐*SlCLV3* lines. In addition, a previous study showed that the application of SlCLE9 and SlCLV3 peptide together onto tomato meristem mutants effectively rescued the phenotype of enlarged meristems (Xu et al., 2015). This finding suggested a possible role of *SlCLE9* in tomato CLV‐WUS pathway and SlCLE9 might compensate SlCLV3 function in regulating *SlWUS* expression.

In addition, we found a moderate upregulation of *SlCLV3* expression in F&IMs and a significant increase of *SlWUS* expression in floral buds at 6 dpi in *lc* mutant (Figure 6). The results were consistent with our hypothesis that the two SNPs present downstream of the 3′ UTR of *SlWUS* might abolish the suppression imposed by tomato AGAMOUS. Intriguingly, *SlWUS* expression was also significantly upregulated in the SYMs of *lc*, which suggested that other unknown mechanisms were involved in the control of *SlWUS* expression through the CArG box in the SYM.

The *in situ* hybridization results confirmed the expression changes of *lc* and *fas* at the tissue level. *SlCLV3* signal was weaker in lines carrying *fas* after 3 dpi, while the *SlCLV3* signal was stronger and more persistent in lines carrying *lc*. In addition, the *SlWUS* expression domain expanded laterally in lines carrying one or both mutations at 2–3 dpi, when sepal primordia appeared. This correlated with the enlarged FM size at 3 and 4 dpi in *lc*,* fas* and *lc*/*fas* NILs (Figure 3). Nevertheless, the expansion of *SlWUS* signals was not observed in single and double mutants after 4–5 dpi, when petal and stamen primordia were initiated. This might be due to the presence of unknown suppressors that function on or before 4 dpi. Since the expression of TAG1 was not likely to lead to downregulation of *SlWUS* at the earliest timepoints, it was conceivable that Solyc05 g015750 (TM5 closely related to SEP3) and Solyc02 g089210 (closely related to AP1) led to downregulation of *SlWUS* expression. The latter two MADS box proteins were expressed early in tomato floral development and were upregulated in the mutants. In *SlCLV3* RNAi lines, *SlWUS* expression domain was strongly expanded and the expansion persisted to the late stages of floral development, consistent with the phenotype observed in Arabidopsis *clv3* null mutant (Brand et al., 2000; Schoof et al., 2000).

Although a slight expansion of *SlWUS* expression domain in floral buds was observed in all mutants at 2–3 dpi (Figure 5), differential *SlWUS* expression was not detected between genotypes at 2 dpi from the RNA‐seq results. This could be due to a similar rate of increase in FM enlargement and the expansion of *SlWUS* expression domain in mutants. Furthermore, although we found a relatively weak spatiotemporal *SlCLV3* expression signal at 3–4 dpi in *fas* and *lc*/*fas* NILs in comparison to the wild type (Figure 5), the expression of *SlCLV3* in *fas* and *lc*/*fas* NILs did not show a corresponding decrease at 4 dpi based on the RNA‐seq results. This again might be due to the increase of total cells that expressed *SlCLV3*, albeit the expression level was lower on a per cell basis.

Unlike Arabidopsis, in which *CLV3* is expressed in the L1, L2 and L3 layer (Fletcher et al., 1999), we found that *SlCLV3* was absent from L1 and its expression was above and partially overlapped with the *SlWUS* expression domain in tomato. Similar results were also observed in soybean, in which *GmCLV3* was absent in L1‐L3 layer and its expression domain overlapped with *GmWUS* below the L5 layer in the SAM (Wong, Singh, & Bhalla, 2013). Together, these findings raise the possibility that the CLV‐WUS meristem regulatory mechanism has somewhat diverged across different species.

In summary, our results imply that *lc* and *fas* mutations cause an expansion of the *SlWUS* expression domain and delay the termination of *SlWUS*, resulting in the production of larger FMs and more floral primordia.

### 3.2 Genes responding to *LC* and *FAS* expression dynamics during early floral development

Compared to a previous report using a DEX inducible system to identify WUS target genes in Arabidopsis (Busch et al., 2010), 91 out of 675 DEGs were found in both studies, including *STM* and *AINTEGUMENTA* (*ANT*) (Data S2). In addition, 133 out of the same 675 DEGs were differentially expressed in the RNAi‐*SlCLV3* line (Data S3). These common DEGs were potential candidates to study conserved mechanisms involved in organogenesis and floral development across different plant species. Identifying DEGs shared by *lc*,* fas* and *lc*/*fas* could potentially help to narrow down genes acting downstream of *SlWUS* and *SlCLV3*. It is hypothesized that the lower expression of *SlCLV3* in *fas* and the higher expression of *SlWUS* in *lc* would result in a significant number of common DEGs. To our surprise, the percentage of overlapping DEGs between *lc* and *fas* was low (5% ~ 36%) (Figure S6, Data S4). This might be because *lc* is a weak allele, having only a minimal impact on locule number in the wild type tomato background. Therefore, the detection of DEGs associated with floral development in *lc* might be limited. Or it could be due to the pleiotropic role of CLV3. CLV3 belongs to the CLE small peptide family, which is also involved in plant‐microbe interaction, vascular development and long‐distance signal transduction (Betsuyaku, Sawa, & Yamada, 2011; Kucukoglu & Nilsson, 2015). Strabala et al. (2006) showed that the ectopic expression of *CLV3* causes anthocyanin accumulation in Arabidopsis. Our results also show that the DEGs overlapping between *fas* and *lc*/*fas* (*fas*‐secondary) were enriched in lignin metabolism, flavonoid biosynthesis, and response to environmental stimuli (Figure S6). Together, these results indicated that *SlCLV3*, in addition to its involvement in maintaining the meristem cell population with *SlWUS*, might be involved in other developmental processes as well.

We also observed a trend of decrease in the number of overlapping genes as floral buds developed further (Figure S6). Because whole flower buds were used in this study (Figure S1b), the growing mass of sepals, petals and stamens might dilute the meristem‐specific transcripts and therefore hinder the detection of DEGs between genotypes.

### 3.3 Novel mechanisms underlying LC‐FAS mediated control of meristem development revealed by co‐expressed gene clusters

The transcriptional network controlling tomato meristem development is not fully unexplored. To reveal potential players participating in *LC‐* and *FAS‐* mediated meristem and floral development programs, a time‐course RNA‐seq gene expression profiling was conducted. From the cluster analysis, we uncovered a cluster enriched with genes related to microtubule motor activity (Figure 7, Data S1). This group of genes only showed higher expression in *lc* compared to the wild type and *fas*, especially in the SYM (Figure S7, Data S1). It is possible that this group of genes is activated by CLV‐mediated MAPK signaling cascade (Betsuyaku, Takahashi et al., 2011), as CLV activity is elevated in *lc*. In Arabidopsis, MAPK cascade targets various TFs involved in a plethora of developmental, defense, and stress responses (Popescu et al., 2009). The tobacco MAPK cascade, positively regulates cytokinesis by phosphorylating NtMAP65‐1, a microtubule‐associated protein (Sasabe et al., 2006). In our results, we also found an elevated expression of a tomato microtubule‐associated protein gene *MAP65‐1a* (Solyc11 g072280) in *lc*.

The identification of a cluster of genes involved in phytosterols and BRs synthesis indicates their putative roles in meristem function during tomato floral development. Genes in this cluster were expressed at lower levels in *fas* and *lc*/*fas*, indicating that the BR level might also be lower in the meristem of *fas* and *lc*/*fas* (Figure 7, Data S1). Phytosterols are the precursors of BRs and the homeostasis of BRs is controlled by a feedback regulatory loops (Vriet et al., 2013). A previous study demonstrates that phytosterols have a BR‐independent role in controlling and activating signals for plant development (He et al., 2003). The sterol biosynthesis activities appear to be highly localized to the meristematic region. For example, the tobacco squalene synthase (SQS) enzyme activity is predominantly detected in SAM (Devarenne, Ghosh, & Chappell, 2002). Arabidopsis 3‐hydroxy‐3‐methylglutaryl coenzyme A reductase 2 (*HMG2*) gene is expressed in SAM and floral tissues (Enjuto, Lumbreras, Marín, & Boronat, 1995). In addition, Arabidopsis *FACKEL* gene is set involved in embryonic patterning and meristem programming (Jang et al., 2000).A Although we propose that sterol biosynthesis in meristematic regions is affected in *fas*, we cannot rule out the possibility that the linked genes located within the introgressed *fas* inversion are the cause of the differential expression.

Brassinosteroids are growth promoting hormones in general and the BR contents are maintained at a low level in the meristem, particularly in the organ boundary (Hepworth & Pautot, 2015). The KNOX genes, such as *STM*, maintain the identity of meristem and boundary in the SAM by suppressing the BR levels and directly activating genes involved in boundary formation (Bolduc et al., 2012; Johnston et al., 2014; Spinelli, Martin, Viola, Gonzalez, & Palatnik, 2011; Tsuda, Kurata, Ohyanagi, & Hake, 2014). In Arabidopsis, BR biosynthesis mutant, *det2*, and BR membrane‐bound receptor mutant, *bri1*, cause extra carpel formation (Gendron et al., 2012). Together, these findings raise the possibility that higher expression of *SlWUS* and tomato *STM* in the FMs of *lc*,* fas* and *lc*/*fas* suppressed sterol/BR biosynthesis, thereby triggering the formation of extra boundaries and floral organs.

In summary, our in situ hybridization and RNA‐seq analyses have captured the dynamics of gene expression in vegetative meristem, floral meristem, and young floral buds in *lc* and *fas*. These results have provided useful information for the future study of important developmental questions, such as the link between the meristem regulation and floral organ determinacy. These results can also be integrated with other large‐scale datasets at various levels to decipher the regulatory network in meristem development, and provide predictive models in improving fruit traits.

## 4 MATERIALS AND METHODS

### 4.1 Plant materials and near‐isogenic line (NIL) development

*Solanum pimpinellifolium* accession LA1589 seeds were obtained from *Tomato* Genetics Resource Center (http://tgrc.ucdavis.edu/). Mature fruits from LA1589 typically contain two locules and are about 1 cm in diameter. Both *Solanum lycopersicum* cv. Orange Strawberry and Yellow Stuffer seeds were obtained from Tomato Growers Supply Company. Orange Strawberry contains the *lc* and *fas* mutant alleles and bears large fruits with 14 locules on average. Yellow Stuffer bears large fruits with 3.6 locules on average and only carries the *lc* allele. The NILs carrying the mutant alleles were derived from repeated backcross to the wild species *S. pimpinellifolium* accession LA1589. After six backcross generations to introgress each locus separately, the *lc* and the *fas* lines were crossed to one another to create the double NIL. To further reduce the size of the introgression regions at both loci, we made three more backcrosses to LA1589 and identified close recombination breakpoints, followed by two more self‐pollinated generations to generate final BC_9_F_2_ population (family 13S133) (Figure S8). During this selection, *lc* and *fas* loci were maintained in the heterozygous state, while the surrounding loci were selected to be homozygous wild type and selected for recombinants around the genes. Three NILs, *lc*,* fas*,* lc/fas* and the wild type (WT), were created from the BC_9_F_2_ population. The primers used to select recombinants are listed in Table S3.

The size of the introgressed segment varied for each locus (Figure S9). For *lc*, the region was approximately 184 kb, from 47,014 to 47,198 kb on Chr.2 (SL2.50). For *fas*, the introgression size was about 351 kb, from 54,842 to 55,193 kb on Chr.11 (SL2.50). The *fas* mutation was caused by a large inversion (294 kb) which limited the ability to narrow down the region further in this NIL.

### 4.2 Cloning of the RNAi‐*SlCLV3* constructs

To reduce the expression of *SlCLV3* in wild type tomatoes, a hairpin RNAi construct was created using the pKYLX80 vector similar to the method described in Siminszky, Gavilano, Bowen, and Dewey (2005). A gene‐specific fragment of 355 bp was amplified from *SlCLV3* with the following primer pairs: CRF1: 5′‐AATTCTAGAAGCTTTCAATCTCT TTGTCTTGCTGA‐3′ and CRR1: 5′‐ATGGAGCTCTCGAGATGAA ACCATATACTACCCT‐3′. The amplified product was digested with *HindIII*/*XhoI* and *SacI*/*XbaI* to construct sense and antisense fragments flanking the 151 bp region of soybean ω‐3 fatty acid desaturase (FAD3) intron (Figure S10). Next, both digested fragments were inserted into vector pKYLX80. The resulting *EcoRI*‐*XbaI* fragment from pKYLX80 containing the *CaMV35S2* promoter, *SlCLV3* sense hairpin‐stem*, FAD3* intron and *SlCLV3* antisense hairpin‐stem was subcloned into binary vector pKYLX71 between TL border and the RBCS subunit terminator to produce the final RNAi‐ *SlCLV3* construct, named pRNAi‐CR. The pRNAi‐CR was stably transformed into *S. pimpinellifolium* accession LA1589. We selected two independent T0, pRNAi‐CR4 and pRNAi‐CR9, which were shown to contain six and one copy of the transgene respectively, and were further evaluated by phenotypic and expression analysis in the T1 generation. Target specificity of this RNAi experiment was examined through BLASTN using Tomato genome cDNA database (SL3.20) with the designed hairpin‐stem sequence (Figure S11). Comparisons of the expression level of all the *SlCLE* gene families between the wild type and RNAi‐*SlCLV3* lines in tomato FM and 2dpi floral buds are shown in Figure S12.

### 4.3 Morphological analysis

#### 4.3.1 Inflorescence branching, floral organ number and fruit locule number counts

Five to six plants were selected from the seedlings generated by family 13S133 for each genotypic class (*lc*,* fas*,* lc*/*fas* and the wild type) and transplanted in 1‐gallon pots in the greenhouse. For the transgenic T1 lines, three to seven plants were used. Inflorescence branching was evaluated on 40 inflorescences per plant. In addition, 40 flowers at anthesis were collected per plant to evaluate sepal, petal and stamen number. To evaluate locule number, 40 ripe representative fruits were collected from each plant, and locule number was counted in cut fruits.

#### 4.3.2 Fruit weight and dimension analysis

For fruit weight analysis, 20 ripe representative fruits per plant were selected and weighted. For fruit dimension analysis, eight to ten fully mature fruits from each of the genotypes were cut horizontally and scanned. Tomato analyzer 3.0 (Rodríguez et al., 2010) was used to analyze the scanned images for fruit perimeter and area following the instructions (http://vanderknaaplab.uga.edu/tomato_analyzer.html).

#### 4.3.3 Morphological analysis of inflorescence structure and meristem size measurement

The first young inflorescences of *lc, fas, lc/fas* NILs and the wild type were collected in the greenhouse and immediately placed in ice‐cold RNA*later* (QIAGEN) to preserve the tissue structure. The inflorescences were imaged using an Olympus SZH10 stereo‐microscope and Olympus DP‐10 digital camera. For the meristem size measurement, paraffin slide sections were made the same way as described in in situ hybridization procedures. Paraffin slide sections were rehydrated through ethanol series and stained with 1% Toluidine Blue (Sigma‐Aldrich). Stained tissues were further dehydrated through ethanol series and finally mounted with Cytoseal 60 (Thermo Scientific). Images were taken under a fluorescence microscope and meristem size was measured using ImageJ software (NIH). The width of floral meristems was measured along a line between two sepal and petal primordia in floral buds at stage 3 and 4 days post floral initiation (dpi), respectively. For each genotype and time point, at least five meristems were measured. A two‐tailed *t*‐test was performed for statistical analysis.

#### 4.3.4 Statistical analysis

Analysis of variance (ANOVA) and Tukey's mean separation tests (HSD) were performed using the average of 20–40 measurements from each plant except for fruit size dimension, in which an average of eight fruits were used. Comparisons were made using the average per plant and 3–7 plants per genotype. Epistasis between the two loci was determined using two‐way ANOVA with the following model: *Y*_*ijk*_ = μ + *a*_*i*_ + *b*_*j*_ + *ab*_*ij*_ + ϵ_*ijk*_, in which *a* and *b* represented the effect of *lc* and *fas*, whereas *ab* was the interaction factor. In addition, *i* represented the *i*’th allele at *lc*,* j* represented the *j*’th allele at *fas*, while *k* represented the number of all plants used in the analysis. To estimate how *lc* and *fas* contributed to trait variance, dominance‐to‐additive variance ratio (d/a) was calculated using the following equation: d/a = (2Aa‐AA‐aa)/(AA‐aa). AA and aa represented the phenotypic effects caused by homozygous derived and wild type alleles, respectively. Aa represented the phenotypic effect caused by heterozygous alleles.

### 4.4 In situ hybridization to determine *LC* and *FAS* expression in floral meristems

RNA in situ hybridization was performed with digoxignin‐labeled RNA using by Wu, Xiao, Cabrera, Meulia, and van der Knaap (2011) with minor modifications. To generate the RNA probes, full‐length *SlCLV3* (Solyc11 g071380) and *SlWUS* (Solyc02 g083950) cDNA was amplified from M82 cDNA using Phusion Taq (Invitrogen) and ligated into the pSC‐A‐amp/kan vector containing T7 and T3 promoter (provided by Lippman's lab, CSHL). Clones pSL‐CLV3‐1 and pSL‐CLV3‐4 were generated to make *SlCLV3* antisense and sense probes, respectively. Clone pSL‐WUS‐4 was used to make both *SlWUS* sense and antisense probes. Depending on the orientation of the insert, T7 or T3 RNA polymerase was used to transcribe sense or antisense RNA.

Young inflorescences were fixed with ice‐cold 4% (w/v) paraformaldehyde and vacuum infiltrated with a pressure of 25–28 in Hg for 20–30 min until the samples had sunken. Samples were dehydrated through ethanol series, followed by histoclear replacement. Paraffin wax (Polyscience) was used for sample embedding with at least six fresh exchanges for 3 days. Microtome sections were taken to obtain 10 μm thick ribbons. Slides were rehydrated in an ethanol series followed by Proteinase K digestion and acetylation. The hybridization reactions were conducted at 55°C overnight with the gene‐specific DIG‐labeled probes. Excess probes were washed off with saline‐sodium citrate buffer (SSC buffer) and slides were blocked with blocking reagent (Roche). To detect the signal, slides were incubated with alkaline phosphatase‐conjugated antibody (anti‐DIG‐AP Fab fragments, Roche) at room temperature for 2 hr. Non‐specific binding of antibody was washed three times with BSA buffer for 1.5 hr each. Finally, Western Blue (Promega) was applied to each slide and incubated overnight in dark at room temperature for the color reaction. Images were taken under the fluorescence microscope (Leica) equipped with a digital camera in Molecular and Cellular Image Center in OARDC.

### 4.5 Tissue collection and RNA extraction

For each genotype, approximately 300 first and second inflorescences with only 3–4 visible floral buds were collected from 5 to 7‐week old plants that were sown over a 6‐week window. For each replicate, tissues were collected daily between 2 and 3 p.m. During the collection, all inflorescences were immediately immersed in ice‐cold RNA*later* (QIAGEN) solution at five times the volume of the sample in order to prevent RNA degradation. After sample collection, vacuum infiltration was applied until tissues sunk to the bottom of RNA*later* solution. The tissues were then stored in RNA*later* at −80°C. Five different developmental stages including sympodial shoot apical meristem (SYM), floral and inflorescence meristems (F&IM), 2 days post floral initiation (dpi), 4 and 6 dpi floral buds were collected from each genotype with three replicates. For the dissection, meristems and buds of different developmental stages were isolated using forceps under a dissection microscope and immediately put into a 1.5 ml eppendorf tube in 1 ml of fresh RNA*later* that was kept on ice. The samples were stored at −80°C prior to the RNA extraction. Prior to RNA extraction, the precipitated RNA*later* crystals need to be dissolved by occasionally shaking of the eppendorf tube at room temperature several times. The RNA*later* reagent was further removed using a fine‐tip drawn‐out glass pipette. RNA extraction was conducted using Trizol^®^ (Invitrogen Inc.) following manufacturer's recommendation. The quality of RNA in each sample was examined using Agilent Bioanalyzer prior to RNA library preparation. Samples with a total RNA amount of 1–2 μg were used for subsequent RNA library preparation.

### 4.6 3′ Tag RNA‐seq library preparation

3′ Tag RNA‐seq, a low‐cost RNA‐seq alternative, was adapted to our study. Meyer, Aglyamova, and Matz (2011) presented a 3′ Tag RNA‐seq approach that solely focused on sequencing the 3′ end of mRNA. This 3′ Tag RNA‐seq method requires only 5 million reads per sample, which can significantly reduce the cost for sequencing per sample by allowing a higher degree of multiplexing. RNA‐seq libraries of approximately 300 bp fragments were prepared following the 3′‐Tagseq protocol of Meyer et al. (2011) with little modifications as per directions from Dr. Thomas Juenger, The University of Texas at Austin. In total, sixty libraries were made, including our four genotypes at five developmental stages, and each with three biological replicates. The mRNA was enriched using Oligo d(T)_25_ magnetic beads (NEB) by following the manufacturer's recommendations. The bound mRNA was eluted from the beads by adding 2× SupertScript II first‐strand buffer (Invitrogen) supplemented with 10 mM DTT. Samples containing eluted mRNA, magnetic beads and first‐strand buffer were incubated at 94°C for 2 min to fragment the mRNA, and immediately placed on ice. Samples with fragmented RNA were placed on the magnetic rack to remove the Oligo d(T)_25_ beads. The supernatant containing fragmented mRNA and first‐strand buffer was transferred to a new tube for first‐strand cDNA synthesis. First‐strand cDNA was synthesized using SupertScript II reverse transcriptase in the presence of 3′ Oligo dT primers and 5′ RNA adaptors with GGG at 5′ end at 42°C for 1 hr. To amplify targeted 3′ end cDNA, AccuPrime Taq polymerase (Invitrogen) was used for the 16 cycles of PCR amplifications. To purify the resulting 3′ end cDNA products, the excess primers, nucleotides, salts and enzymes were removed by Agencourt AMPure XP using 1.8 volume of beads solution. cDNA quantity was measured using the Qubit HS and all samples were diluted to 40 ng in 42 μl total volume. Next, library‐specific barcodes and Illumina universal adaptors were incorporated to each cDNA by five cycles of PCR in 50 μl total volume using AccuPrime Taq polymerase (Invitrogen). Afterwards, six libraries were pooled together. To size select 300–350 bp fragments, 0.7 volume of Agencourt AMPure XP beads to 1.0 volume of sample was used and the supernatant was collected to remove cDNA size larger than 400 bp. Next, 0.85 volume of Agencourt AMPure XP beads to 1.0 volume of sample was used to target cDNA size around 300–350 bp. The bound cDNA was eluted from the beads by adding distilled water. The fragment size and concentrations of the samples were examined using Agilent Bioanalyzer and Qubit HS. Samples with 30 ng total amount were mixed together to create two pools with 30 3′ Tag RNA‐Seq libraries each. The RNA libraries were sent to the Genomic Resources Core Facility at Weill Cornell Medical College for 100 bp single–end sequencing on the Illumina HiSeq2500. The raw data are deposited at NCBI under accession numbers GSE129809 (3′Tag RNA seq) and GSE129901 (whole mRNA seq).

### 4.7 Gene expression analysis

Raw reads were checked for their quality in FASTQC. Total raw reads for each sample ranged between 5 to 10 million (Table S4). Filtering steps were performed using fqtrim program (http://ccb.jhu.edu/software/fqtrim/index.shtm) to remove up to 50% low quality reads with QC score < 20 as well as poly‐A tail contaminations. About 94% of clean reads mapped to ITAG2.4 Released Tomato Genome through Rsubread 3.4 (Liao, Smyth, & Shi, 2013) and 81% of clean reads mapped to annotated genes. Most of the genes were either not expressed or expressed at low levels (Figure S13). Pearson correlations were performed to check the reproducibility between replicate. An average correlation (*r* = 0.99) was obtained which showed a high reproducibility between samples of the same stage and genotype in this experiment. For the sequencing coverage, around 55%–60% of 29,324 tomato reference genes showed at least three mapped reads among all samples. Gene expression levels were normalized using the RPM value (Reads Per* *Million). The significance of differentially expressed genes (DEGs) was determined by linear factorial modeling in DEseq2, of which likelihood ratio test was applied (Clevenger et al., 2017). To identify genes with significant genotype effects using DEseq2 in R, the full model (Genotype + Time) and reduced model (Time) were used to test whether the observed differences in read counts of a given gene between genotypes were significantly larger than the variations between developmental stages and replicates. Similarly, genes with significant genotype by time point interactions were identified form the full model using (Genotype + Developmental stage + Genotype * Developmental stage) as well as the reduced model (Genotype + Developmental stage). For the genotype category, DEGs with adjusted *p*‐value < 0.1 in at least one comparison were selected, whereas the threshold of *p*‐value < 0.001 was used for selecting DEGs with interaction effects. The *p*‐value was adjusted with the default Benjamini‐Hochberg method in DEseq2. The relatively lower stringency (adj. *p* < 0.1) was used as a cutoff in the genotype category because of the weak cis‐regulatory mutant alleles at *lc* and *fas*, which leads to 669 DEGs with significant genotype effect. In addition, because very few DEGs (seven genes) were identified in the interaction category with adj. *p* < 0.1, we objectively selected a *p*‐value cutoff based on *SlWUS* expression, close to the *p*‐value of <0.001. As a result, 13 DEGs with significant interaction effects were identified. Notably, the model we used for identifying genotype effect contained gene variables with genotype × developmental stage effects. Therefore, we identified seven DEGs genes shared by the two methods.

To obtain an overview of enriched Gene Ontology (GO) terms for the 675 DEGs, Arabidopsis homologs of these DEGs were used as inputs in the Cytoscape plug‐in, GlueGO v2.1.6 (Bindea et al., 2009). To identify co‐expressed genes, samples were clustered based on the normalized expression (Z‐score) of the wild type across four developmental stages (F&IM, 2, 4, and 6 dpi). By using the Mfuzz package (Kumar & Futschik, 2007) with fuzzy c‐mean algorithm in R, DEGs were grouped into eight clusters. Furthermore, to identify a core of genes showing similar expression dynamics in mutants within each cluster, expression values from *lc*,* fas* and *lc*/*fas* NILs were Z‐score normalized with WT. The normalized expression values were used to calculate PCCs between gene pairs within each cluster. To select the core co‐expressed genes, the PCC matrix was hierarchically clustered using Ward's method and visualized through heatmap3 in R (Zhao, Guo, Sheng, & Shyr, 2014). DEGs clustered within the hierarchical sub‐group were objectively selected as a sub‐cluster as they were tightly co‐expressed among WT and mutants through different developmental stages.

### 4.8 Whole mRNA‐seq sample preparation, library construction, and data analysis

For the whole mRNA‐seq, tissues were collected from the wild type and RNAi‐*SlCLV3* lines at three developmental stages, each with four replicates. The first stage included inflorescence meristems, floral meristems and the youngest floral buds. The second and third stages included floral buds at 4 and 6 dpi, respectively. Plants were grown in two‐gallon pots under natural light supplemented with artificial light (16/8 hr light/dark cycle) in the greenhouse in Wooster, OH, USA, 2013. For each genotype, 100–150 young inflorescences (the largest floral bud was smaller than 0.5 cm) from six individual plants were collected using forceps. For each replicate, tissues were weekly collected during 10 a.m.–12 p.m. in the greenhouse over 4 weeks.

Strand‐specific RNA libraries of approximately 250 bp fragments were prepared following the protocol of Zhong et al., 2011. Briefly, six libraries were barcoded and pooled in one lane. The RNA‐seq libraries were sent to the Illumina HiSeq2000 at Genomic Resources Core Facility at Weill Cornell Medical College for 50 bp single–end sequencing. After filtering low quality reads and de‐multiplexing, the quality of 50 bp raw reads were checked through FastQC (http://www.bioinformatics.babraham.ac.uk/projects/fastqc/). The cleaned raw reads were then mapped to ITAG2.3 Released Tomato Genome through the Tophat2 high throughput short read aligner. Total reads from sense strand for each sample are about 20–30 million. Gene expression levels were normalized using RPKM (Reads Per Kilobase per Million) for whole mRNA‐seq.

### 4.9 Phylogenetic analysis

Phylogenetic tree analysis was performed to assigned nine differentially expressed kinesin genes to one of 10 plant kinesin families. To retrieve the kinesin protein sequences in tomato, full‐length protein sequences were downloaded from the International Tomato Annotation Group release 3.20 predicted proteins (ITAG 3.20) (http://solgenomics.net/). To retrieve the Arabidopsis kinesin proteins, we selected kinesin genes based on Zhu and Dixit (2012) and downloaded their sequence from The Arabidopsis Information Resource (TAIR) database. Sequences of maize kinesin proteins were retrieved from Maize Genetics and Genomics Database (MaizeGDB) website (https://www.maizegdb.org/). Kinesin proteins from other organisms were also selected based on previous analyses reported on the online website (https://labs.cellbio.duke.edu/kinesin/index.html) created by Liz Greene, Steve Henikoff and Sharyn Endow. The Multiple Expectation Maximization for Motif Elicitation (MEME) tool (Bailey & Elkan, 1994) was used to define the conserved motifs in tomato kinesin genes. The multiple sequence comparison by log‐ expectation (MUSCHEL) algorithm implemented in the Molecular Evolutionary Genetics Analysis program (MEGA, version7.0) was used to perform multiple sequence alignment with default settings (Kumar, Stecher, & Tamura, 2016). Phylogeny tree was constructed using the neighbor‐joining method with nucleotide *p*‐distance and 1,000 bootstrap replicates.

## CONFLICT OF INTEREST

The authors declare no conflict of interest associated with the work described in this manuscript.

## AUTHOR CONTRIBUTIONS

YHC and ZH performed the research; YHC contributed to new analyses tools and analyzed the data; JCJ and EvdK supervised the research and writing; EvdK designed the research; YHC wrote the manuscript with revisions from JCJ and EvdK. All authors approved the manuscript.
