# Supplementary figures and images for: Tomato locule number and fruit size controlled by natural alleles of lc and fas
Source: Plant Direct. 2019 Jul 3;3(7):e00142. doi: 10.1002/pld3.142 (PMC6607973; doi:10.1002/pld3.142)

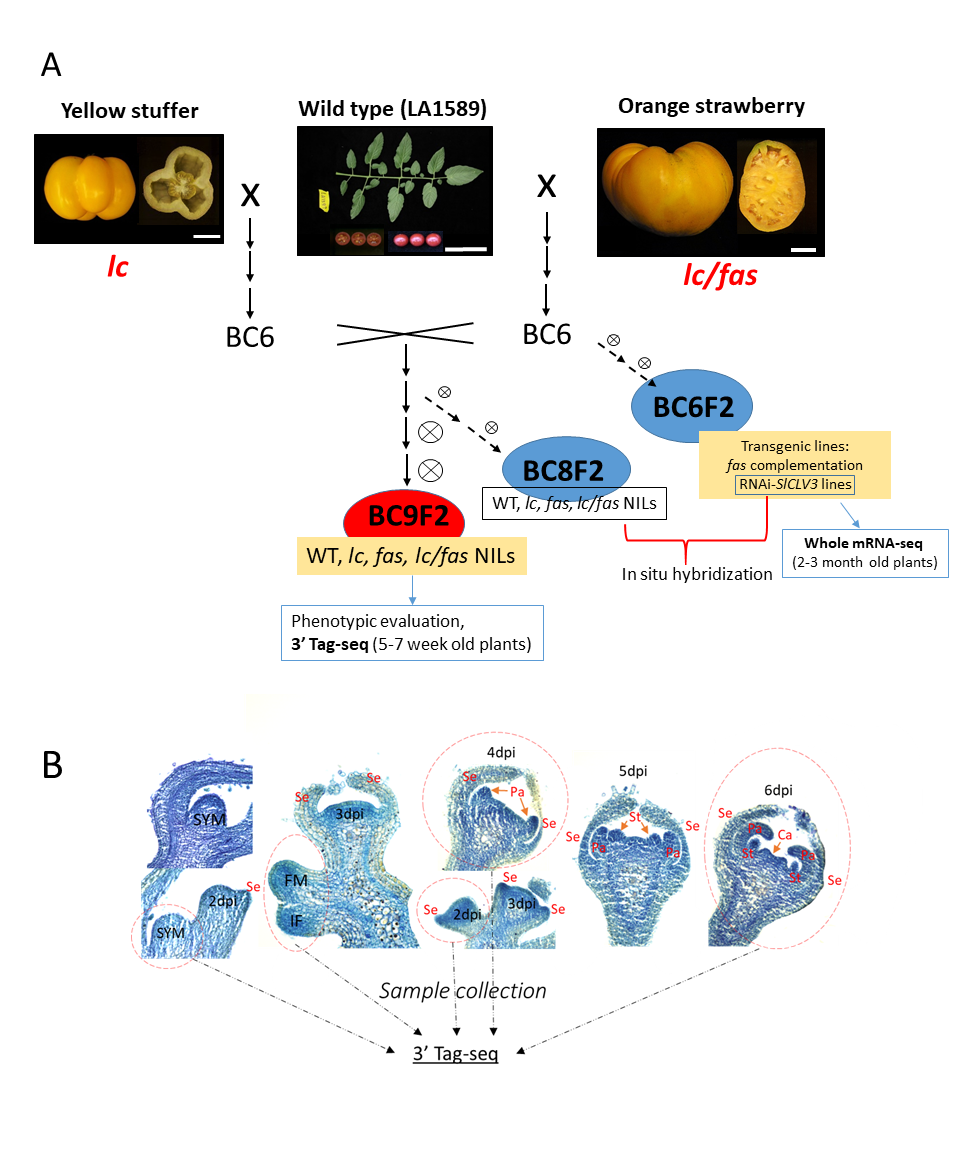

Supplement: Supplementary file 1 [file PLD3-3-e00142-s001.tif]

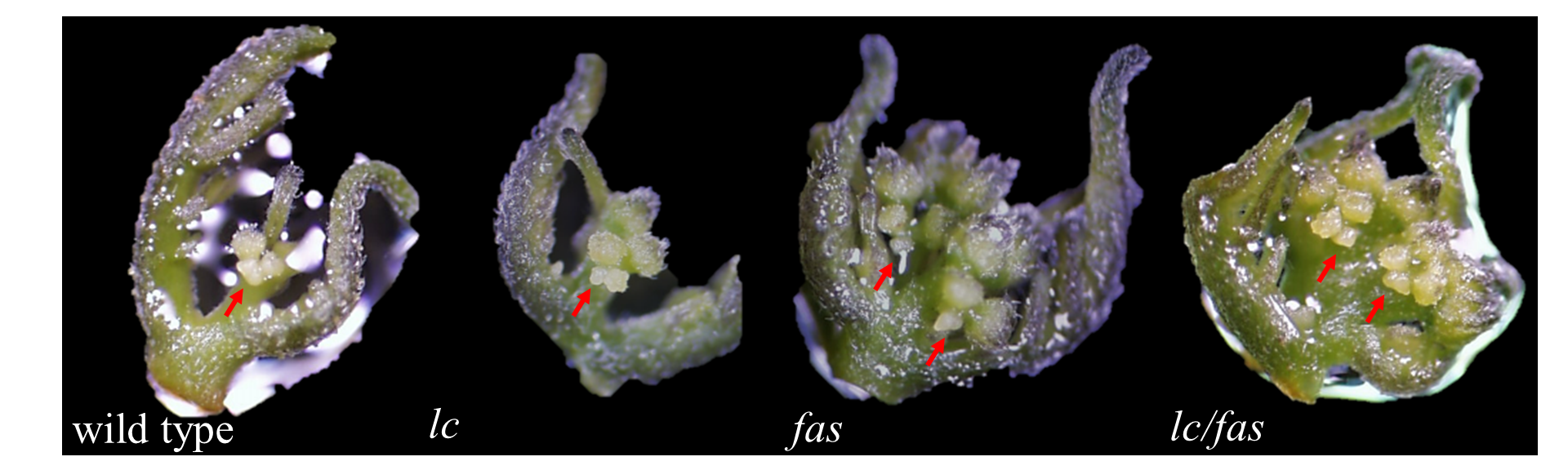

Supplement: Supplementary file 2 [file PLD3-3-e00142-s002.tif]

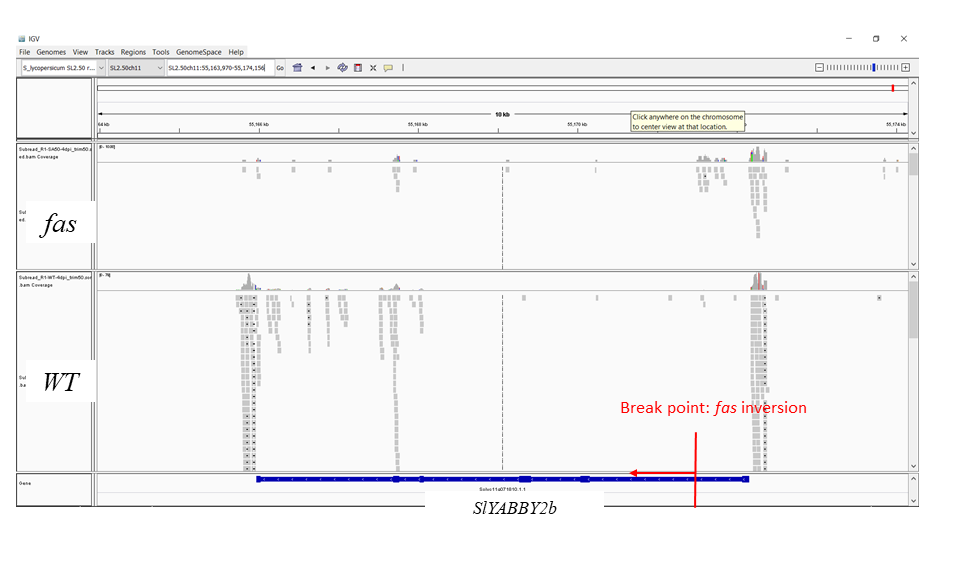

Supplement: Supplementary file 3 [file PLD3-3-e00142-s003.tif]

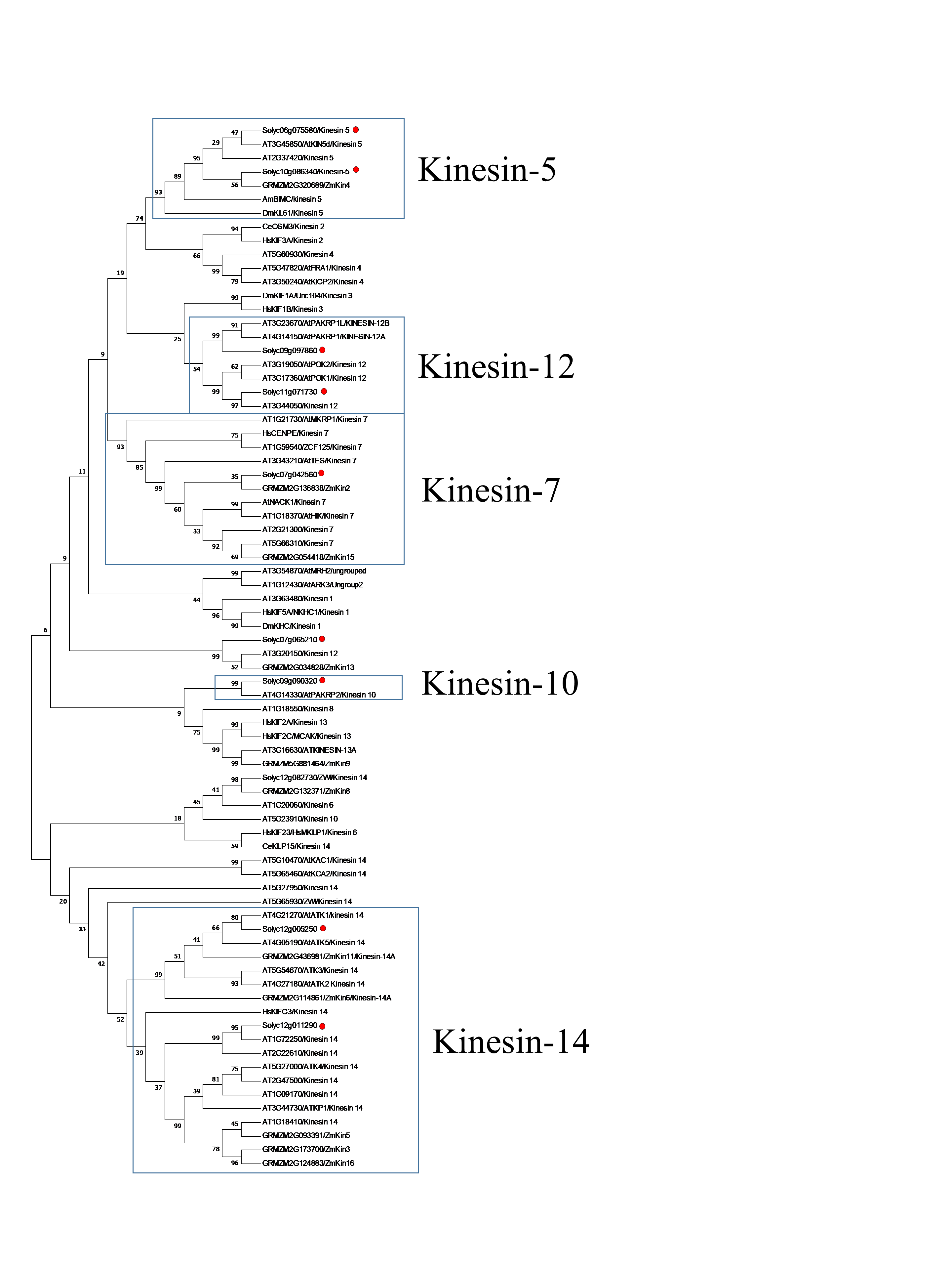

Supplement: Supplementary file 4 [file PLD3-3-e00142-s004.tif]

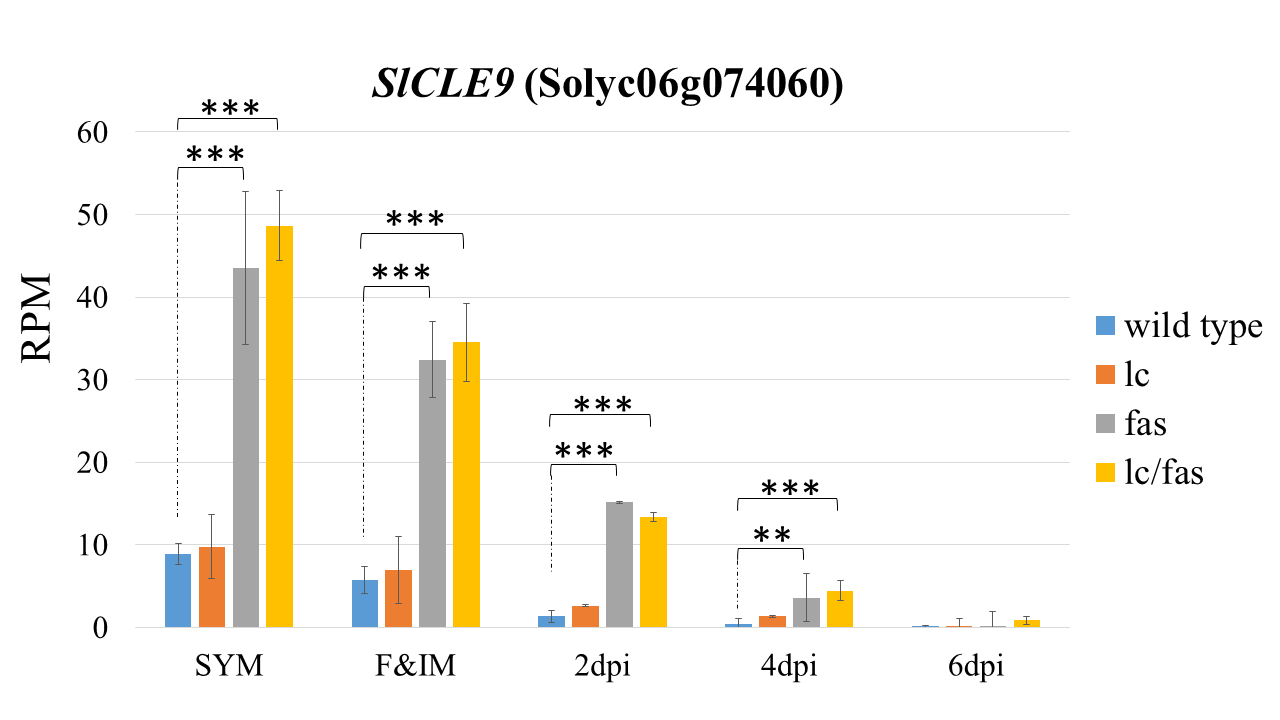

Supplement: Supplementary file 5 [file PLD3-3-e00142-s005.tif]

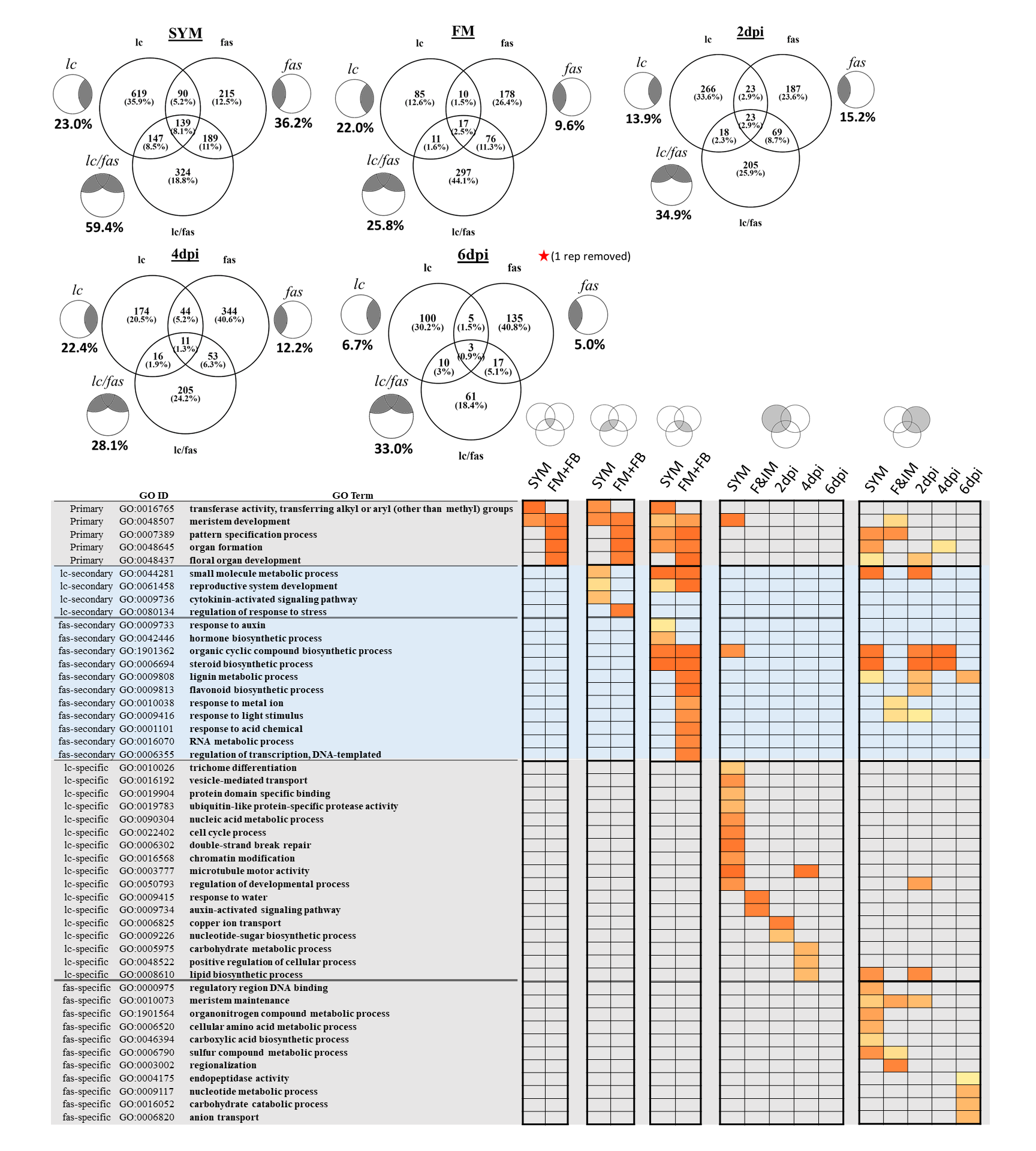

Supplement: Supplementary file 6 [file PLD3-3-e00142-s006.tif]

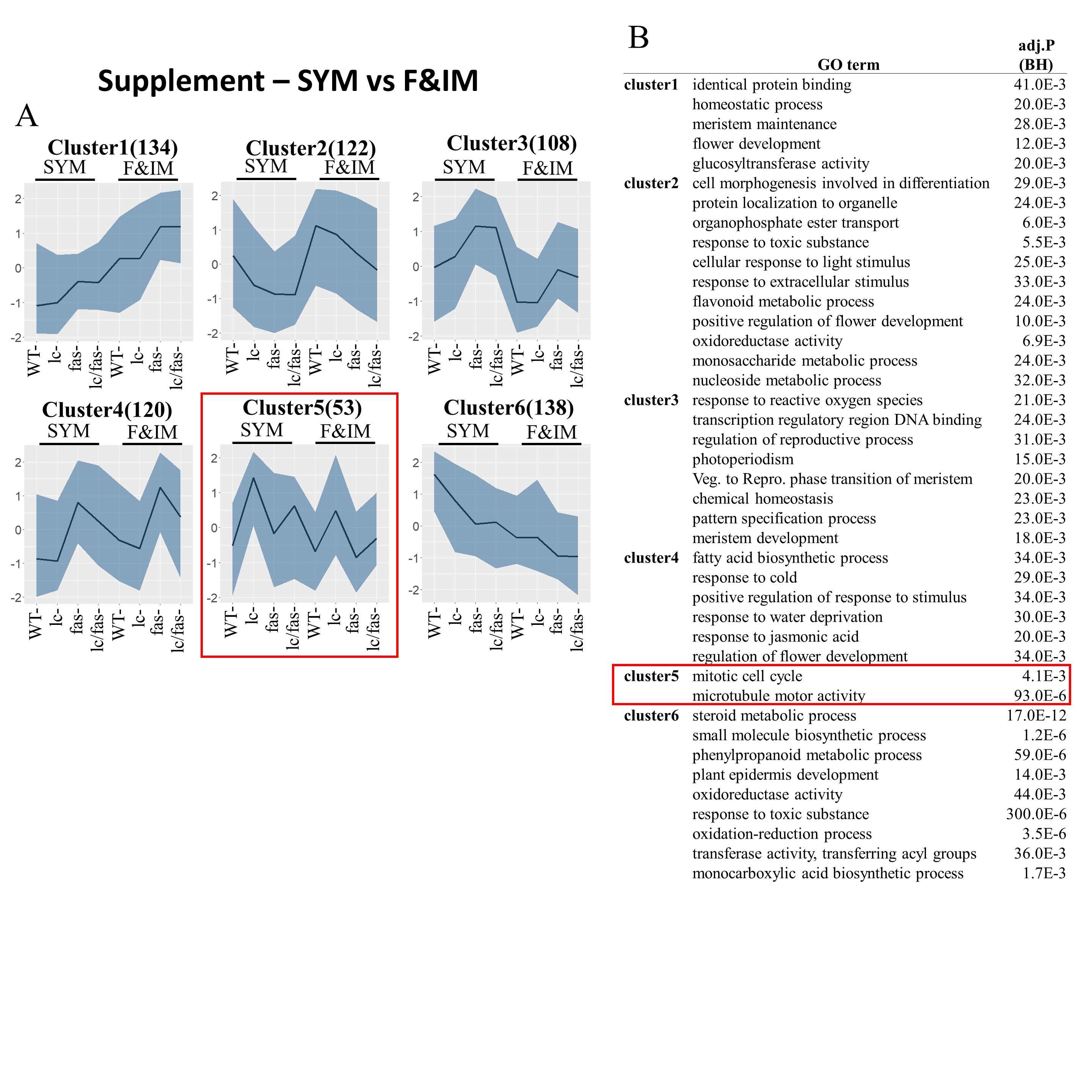

Supplement: Supplementary file 7 [file PLD3-3-e00142-s007.tif]

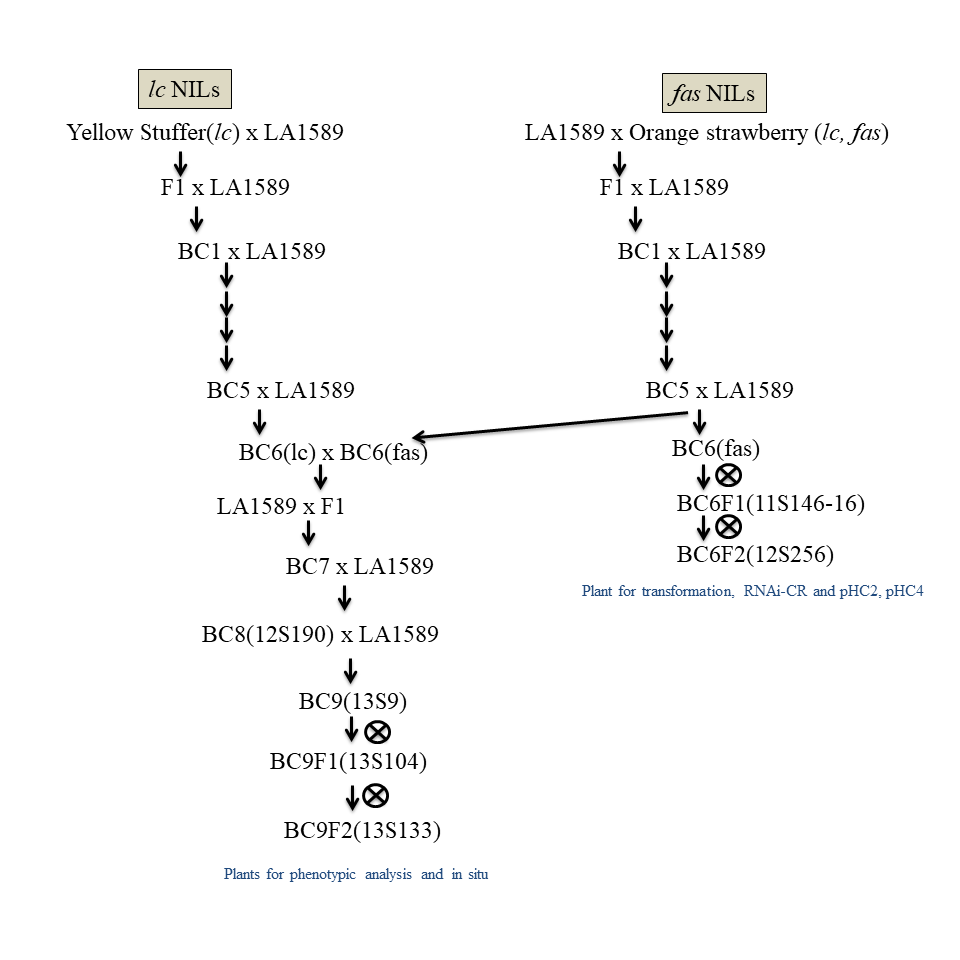

Supplement: Supplementary file 8 [file PLD3-3-e00142-s008.tif]

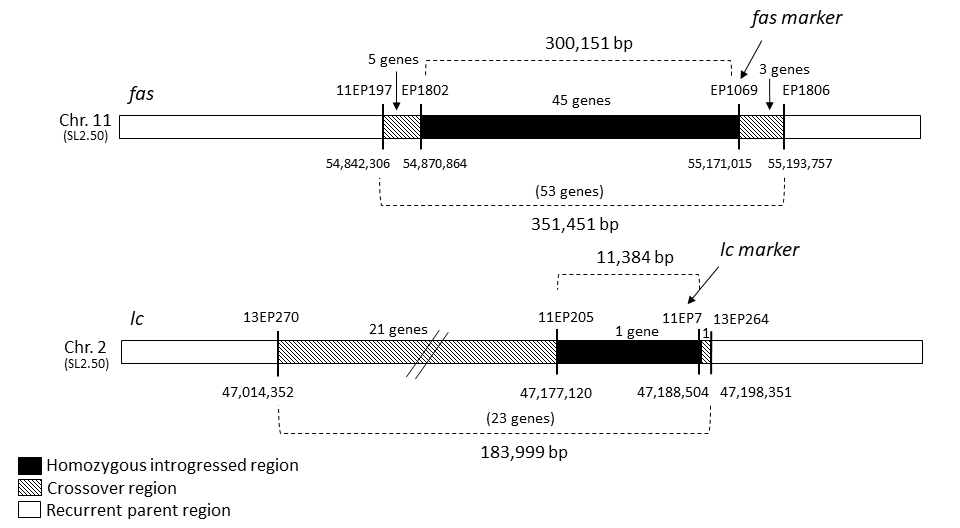

Supplement: Supplementary file 9 [file PLD3-3-e00142-s009.tif]

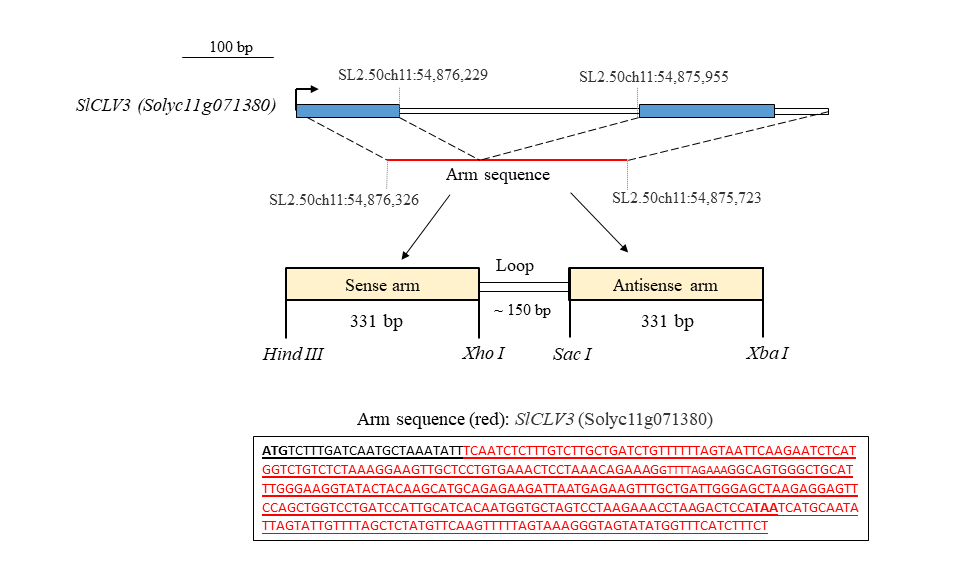

Supplement: Supplementary file 10 [file PLD3-3-e00142-s010.tif]

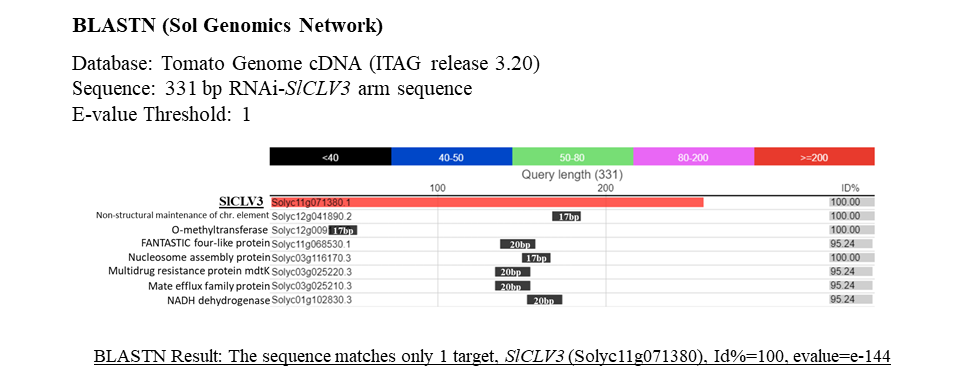

Supplement: Supplementary file 11 [file PLD3-3-e00142-s011.tif]

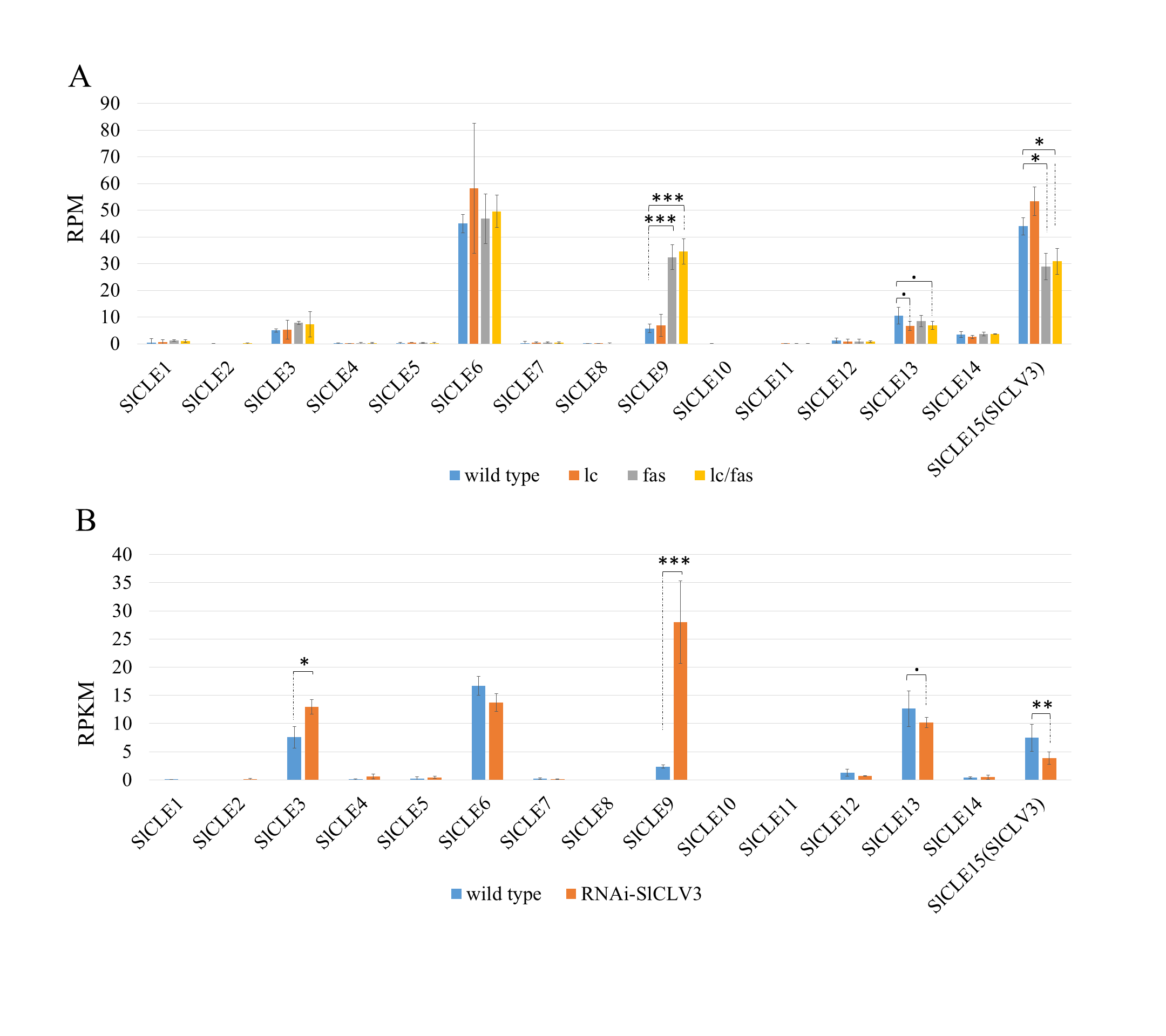

Supplement: Supplementary file 12 [file PLD3-3-e00142-s012.tif]

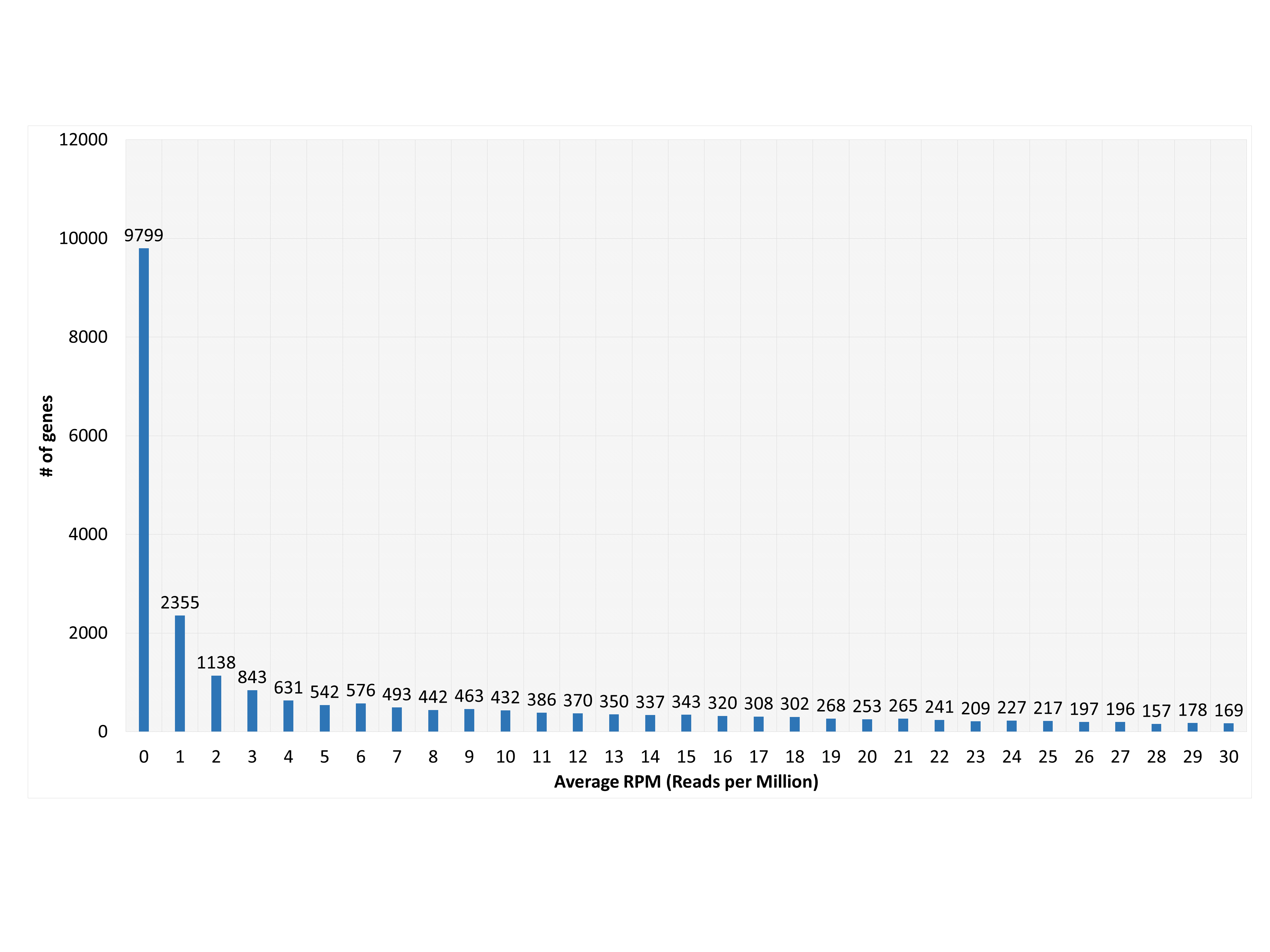

Supplement: Supplementary file 13 [file PLD3-3-e00142-s013.tif]
